# The JNK Pathway Is a Key Mediator of *Anopheles gambiae* Antiplasmodial Immunity

**DOI:** 10.1371/journal.ppat.1003622

**Published:** 2013-09-05

**Authors:** Lindsey S. Garver, Giselle de Almeida Oliveira, Carolina Barillas-Mury

**Affiliations:** Laboratory of Malaria and Vector Research, National Institute of Allergy and Infectious Diseases, National Institutes of Health, Rockville, Maryland, United States of America; Weill Medical College of Cornell University, United States of America

## Abstract

The innate immune system of *Anopheles gambiae* mosquitoes limits *Plasmodium* infection through multiple molecular mechanisms. For example, midgut invasion by the parasite triggers an epithelial nitration response that promotes activation of the complement-like system. We found that suppression of the JNK pathway, by silencing either *Hep*, *JNK*, *Jun* or *Fos* expression, greatly enhanced *Plasmodium* infection; while overactivating this cascade, by silencing the suppressor *Puckered*, had the opposite effect. The JNK pathway limits infection via two coordinated responses. It induces the expression of two enzymes (HPx2 and NOX5) that potentiate midgut epithelial nitration in response to *Plasmodium* infection and regulates expression of two key hemocyte-derived immune effectors (TEP1 and FBN9). Furthermore, the *An. gambiae* L3–5 strain that has been genetically selected to be refractory (R) to *Plasmodium* infection exhibits constitutive overexpression of genes from the JNK pathway, as well as midgut and hemocyte effector genes. Silencing experiments confirmed that this cascade mediates, to a large extent, the drastic parasite elimination phenotype characteristic of this mosquito strain. In sum, these studies revealed the JNK pathway as a key regulator of the ability of *An. gambiae* mosquitoes to limit *Plasmodium* infection and identified several effector genes mediating these responses.

## Author Summary

The mosquito *Anopheles gambiae* is a major vector of human malaria, a disease caused by *Plasmodium falciparum* parasites that results in more than half a million deaths each year. Several signaling pathways in the mosquito have been shown to mediate the mosquito immune responses to *Plasmodium* infection. In this manuscript we investigated the participation of the Jun-N-terminal kinase (JNK) pathway in mosquito defense responses. We found that JNK signaling is required for mosquito midgut cells to induce expression of two enzymes, HPx2 and NOX5, that mediate epithelial nitration in response to parasite invasion. These reactions modify the parasites and promote activation of the mosquito complement-like system that results in parasite lysis. The JNK pathway also regulates the basal level of expression of TEP1 and FBN9, two key components of the complement-like system that are produced by hemocytes and secreted into the mosquito hemolymph. Our studies revealed that JNK signaling plays a key role for mosquitoes to limit *Plasmodium* infection, making it an important determinant of malaria transmission to humans.

## Introduction

Malaria is a worldwide disease that is highly endemic in Sub-Saharan Africa and causes over half a million deaths annually. The mosquito *Anopheles gambiae* is a major vector of *Plasmodium falciparum*, the parasite responsible for most cases of human malaria in Africa. *An. gambiae* can mount effective antiplasmodial responses by activating several signaling cascades involved in immune regulation, such as the *Imd*, *Toll*, and *STAT* pathways [1]–[4]. Pathway activation leads to the transcription of effector genes that mediate the antiplasmodial mechanism. The thioester-containing protein 1 (TEP1) and the fibrinogen-related protein 9 (FBN9) are important components of the mosquito complement-like system that are produced by hemocytes and secreted into the mosquito hemolymph; they bind to the ookinete surface and mediate parasite lysis [5], [6]. Activation of the *Imd* and *Toll* pathways decreases ookinete survival as parasites come in contact with the mosquito hemolymph by promoting TEP1-mediated lysis [1], [3], [7]. In contrast, the STAT pathway targets a later stage of the parasite, the early oocysts, through a TEP1-independent response [4].

We have recently shown a functional link between midgut epithelial nitration and another mosquito antiplasmodial response that targets the ookinete stage of the parasite, the complement-like system [8]. Ookinete invasion results in extensive damage to the invaded cell [9] and induces a two-step epithelial nitration reaction in which expression of nitric oxide synthase (NOS) is followed by the induction of heme peroxidase 2 (HPX2) and nicotinamide adenine dinucleotide phosphate (NADPH) oxidase 5 (NOX5) [8], [10]. The HPX2/NOX5 system potentiates NO toxicity, enhances nitration, and reduces *Plasmodium* survival. Exposure of ookinetes to these chemical reactions as they traverse the midgut cell modifies them and makes them “visible” to the mosquito complement-like system [8]; however, the immune signaling pathway(s) regulating the midgut epithelial response to infection have not been identified.

**Figure 1:**
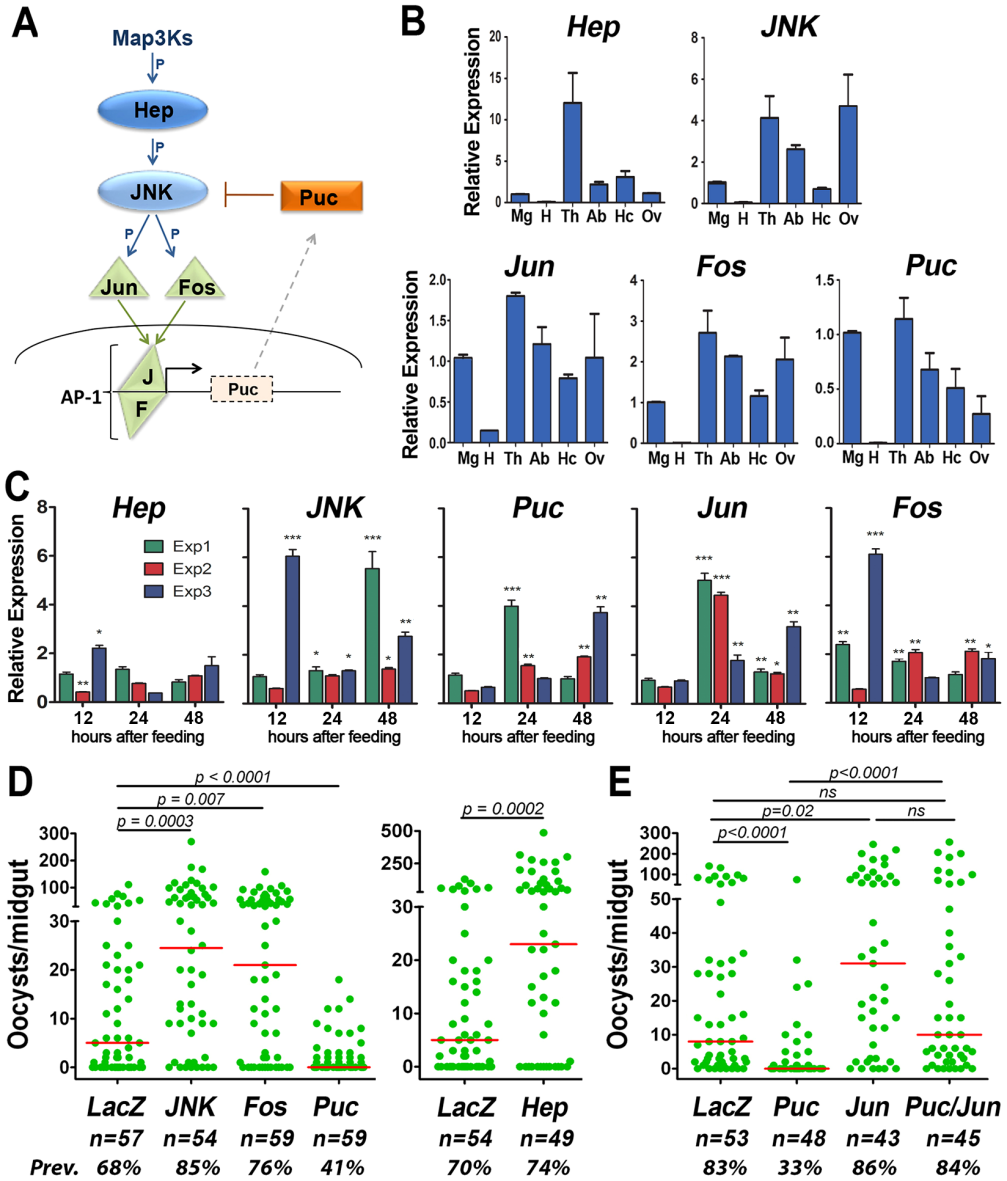
The JNK Pathway and *Plasmodium berghei* infection in *Anopheles gambiae*. (A) Diagram representing the organization of the JNK signaling cascade based on functional studies from vertebrates and *Drosophila*. Five *An. gambiae* orthologs were functionally characterized, including two kinases, hemipterous *(hep)* and c-Jun N-terminal kinase *(jnk)*; a phosphatase, *puc*; and two transcription factors, Jun *(jun)* and Fos *(fos)* (B) Basal mRNA expression of putative genes from the JNK pathway in adult females. *Hemipterous (Hep)*, Jun N-terminal kinase *(JNK)*, *Jun* and *Fos* transcription factors and *puckered (puc)* mRNA levels in different organs of sugar-fed females. Mg, midgut; H, head; Th, thorax; Ab, abdomen; Hc, hemocyte; Ov, ovaries. Expression in different tissues relative to midgut levels, for which the mean was given a value of “1”. Error bars indicate SEM of two biological replicates. (see Table S11 for gene ID numbers and primer sequences) (C) Midgut expression of members of the JNK pathway in response to *Plasmodium* infection in three independent experiments. Ratio of expression in infected/control blood-fed mosquitoes of *Hep*, *JNK*, *Puc*, *Jun* and *Fos* mRNA levels in midguts of mosquitoes from 3 independent experiments (green, red and blue bars). Error bars indicate SEM of two technical replicates. The expression analysis in each biological replicate is shown in Table S3. P-values determined by Student's-T test after log 2 transformation; **, *p*<0.01, ***, *p*<0.001. *, p<0.05; **, p<0.01, ***, p<0.001. (D) Effect of silencing JNK pathway members on *P. berghei* infection. (E) Effect of silencing the transcription factor *jun* alone or and co-silencing *jun* and the negative regulator *puc* on *Plasmodium* infection. For (D) and (E), the green dots represent oocyst counts from individual midguts and the horizontal red bar indicates the median infection level. Groups were compared using the KS, Mann-Whitney and Kruskal-Wallis tests with Dunn's post test (see Table S3). The P-values for the Mann-Whitney tests are shown. Graphs represent samples pooled from three biological replicates with comparable (not statistically different) medians in their *dsLacZ*-injected groups (n = number of midguts).

The JNK pathway is a mitogen-activated protein kinase (MAPK) pathway that is highly conserved from mammals to insects; however, our understanding of the role of JNK signaling in insect immunity is limited. Several orthologs of genes that mediate JNK signaling in vertebrates have been identified in *Drosophila* and *An. gambiae* [11], [12]. The Jun-N-terminal kinase (JNK) is a MAP kinase at the core of this signaling cascade that is activated by a MAPK kinase (*hemipterous*, in *D. melanogaster*) (Figure 1A) [11], [13]–[17]. JNK phosphorylates the Jun and Fos transcription factors, giving rise to a Jun/Fos dimer (AP-1 complex) that activates transcription of target genes (reviewed in [18]). JNK signaling is modulated by *puckered (puc)*, a phosphatase that suppresses signaling by dephosphorylating JNK. Puckered is part of a negative feedback loop, because transcription of *puc* is regulated by the JNK pathway [16], [19], [20].

In *Drosophila*, JNK signaling has been shown to be involved in a wide range of biological processes including embryonic development, apoptosis, stress response, cell proliferation and differentiation, and immunity [18]. The JNK pathway has a great deal of complexity and is known to receive input from multiple upstream genes, yet to be defined in insects, and from lateral inputs from components of other signaling cascades. For example TAK1, a kinase that is part of the *Imd* pathway, can also activate JNK signaling [21]–[23]. It is believed that this complex organization reflects the broad range of responses that are influenced by JNK signaling.

Many different stimuli are known to activate the JNK pathway, including microbial elicitors. In particular, the participation of JNK signaling in antibacterial responses has been well documented in *Drosophila*. Lipopolysaccharide (LPS) is a key elicitor of JNK pathway activity in immune-competent cells and flies [13]–[15], [17], [22], [23], and flies that are deficient in *puc* (and therefore have an overactive JNK pathway output) display increased resistance to Gram^−^ bacteria [19]. In the *An. gambiae* 4a3B cell line, JNK signaling was weakly activated by H_2_O_2_, while LPS elicited a strong response [11]. The response of JNK signaling to LPS has also been observed in human dendritic cells and splenocytes [24].

We have previously shown that JNK regulates expression of several genes that protect *An. gambiae* mosquitoes from oxidative damage, such as oxidation resistance 1 (OXR1), catalase, and glutathione peroxidase [25]. Silencing of these effector genes increased reactive oxygen species (ROS) levels and reduced *Plasmodium* survival. Paradoxically, however, JNK silencing had the opposite effect and enhanced infection, suggesting that—besides the role in ROS balance—JNK may mediate some antiplasmodial response [25]. In this manuscript, we present a detailed functional analysis of several genes that mediate JNK signaling in *An. gambiae* and identify two key mechanisms by which this cascade mediates antiplasmodial immunity. JNK activation induces expression of HPx2 and NOX5, the two enzymes that mediate epithelial nitration in response to ookinete invasion [8]. In addition, JNK signaling regulates the basal levels of expression of TEP1 and FBN9, two effector proteins produced by hemocytes that mediate ookinete lysis [5], [6]. The participation of JNK signaling in the antiplasmodial responses of the *A. gambie* L3–5 strain that has been genetically selected to be refractory to *Plasmodium* infection was also investigated.

## Results

### The *An. gambiae* JNK Pathway Limits *Plasmodium berghei* Infection

Five *An. gambiae* orthologs of genes known to be part of the JNK pathway signaling cascade in *Drosophila* have been identified including two kinases, hemipterous *(hep)* and c-Jun N-terminal kinase *(jnk)*; a phosphatase, *puc*; and two transcription factors, Jun (*jun*) and Fos *(fos)* (Figure 1A,) [11]. These five genes are expressed in the thoraces, abdomens, midguts, hemocytes, and in undeveloped ovaries from sugar-fed mosquitoes (Figure 1B, Table S1). *Jun* is expressed at low levels in the head, but the mRNAs of the other genes could not be detected in this tissue (Figure 1B, Table S1). A notable enrichment of *hep* transcripts in the thorax and of *jnk* in the ovary was observed (Figure 1B, Table S1). The transcriptional response of these five genes to infection with *P. berghei* (rodent malaria parasite) was analyzed in mosquito midguts collected at different times after feeding on either healthy or *P. berghei*-infected mice. A significant increase in *jnk*, *puc*, *jun* and *fos* expression in response to infection was observed between 12–48 hours post infection (hpi). In general, the magnitude and kinetics of the inductions were variable between experiments. *Jun* at 24 and 48 hpi and JNK at 24 hpi had the most consistent inductions that were significant in three independent experiments (Figure 1C, Table S2). *Hep* expression changed the least in response to *Plasmodium* infection (Figure 1C, Table S2). Only a modest increase was observed in one of the replicates at 12 hpi but, in another, the expression was lower after infection than in the uninfected control. Although activation of the JNK pathway involves a cascade of post-translational phosphorylation events, transcription of JNK pathway members has been reported to increase upon *Plasmodium* infection in *Anopheles* and transcriptional activation of JNK at the mRNA and protein level has also been observed in *Drosophila* midguts in response to bacterial challenge [26]–[28]. JNK protein expression was also induced in the mosquito midgut in response to *Plasmodium* infection (Figure S1). This indicates that JNK signaling in infected midguts may be enhanced by increased expression of several components of the cascade.

We confirmed that JNK silencing (Figure S2) enhances *P. berghei* infection (Figure 1D), as previously shown [25]. Furthermore, silencing other genes involved in JNK activation—such as *hep*, *jun*, and *fos* (Figure S2)—also enhanced the intensity of infection, increasing the median number of oocysts by 3.8 to 4.9 fold, relative to the dsLacZ control (Figure 1, D and E, Table S3) (*p*<0.001; Kolmogorov-Smirnov [KS] test). As expected, overactivation of this cascade by silencing *puc* (Figure S2), a phosphatase that normally suppresses JNK signaling, had the opposite effect and greatly reduced the intensity (Figure 1D, Table S3) (*p*<0.001; KS test) and the prevalence of infection from 68% to 41% (*p*<0.005; chi-squared [χ^2^] test). Co-silencing Jun reversed the antiplasmodial effect of silencing *puc* (Figure 1E, Table S3) and increased the prevalence of infection from 33% to 84% (*p*<0.001; χ^2^ test), indicating that Jun is downstream of *puc* and confirming the functional link between these two genes in *An. gambiae*.

### JNK Signaling Activates Midgut Epithelial Nitration

**Figure 2:**
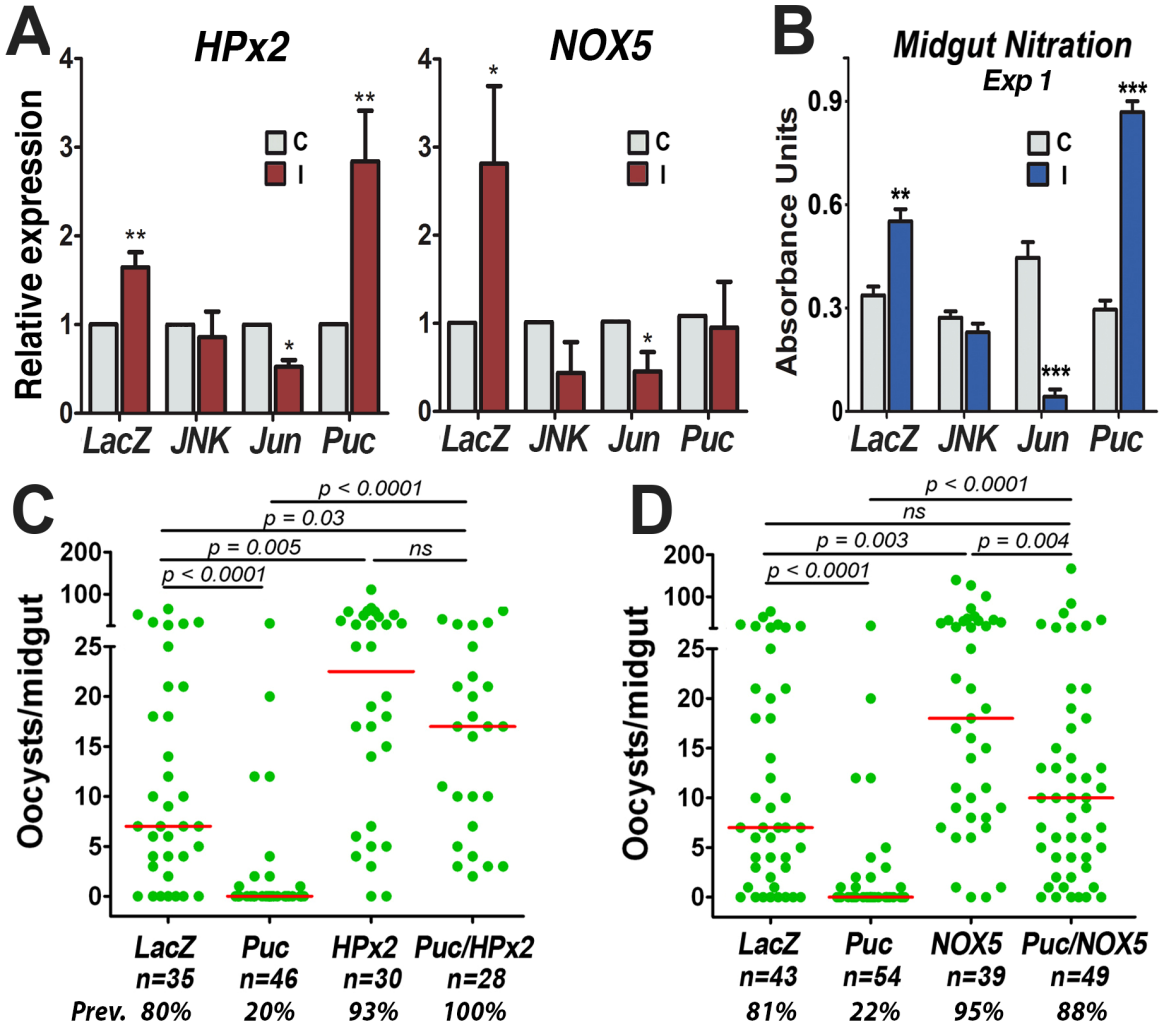
The JNK Pathway and Midgut Epithelial Nitration. (A) Effect of *JNK*, *Jun* or *puc* silencing on the inducible midgut mRNA expression of HPx2 and NOX5 in response to *Plasmodium berghei* infection. C, control mosquitoes fed on a healthy mouse (gray bars); I, infected mosquitoes fed on a *P. berghei*-infected mouse (red bars). Mean expression in infected midguts relative to uninfected blood-fed controls, for which the mean was adjusted to a value of “1”; The bars represent the SEM of three biological replicates from independent experiments (see Table S4). P-values determined by paired Student's-T test after log 2 transformation; **, *p*<0.01, ***, *p*<0.001. (B) Effect of silencing *JNK*, *Jun* or *puc* on infection-inducible *in vivo* midgut nitration. C, control mosquitoes fed on a healthy mouse (gray bars); I, infected mosquitoes fed on a *P. berghei*-infected mouse (blue bars). Graphs represent one of two biological replicates (see Figure S4 and Table S5); error bars indicate SEM of four technical replicates. P-value determined by Student's *t*-test; **, *p*<0.01, ***, p<0.001. (C and D) Effect of co-silencing *hpx2* (C) or *nox5* (D) on the phenotype of silencing the negative regulator *puc*. Green dots represent oocyst counts in individual midguts; horizontal red bar indicates median infection intensity. P-values were determined by Mann-Whitney test; ns, not significant. Graphs represent data from three biological replicates with comparable medians in their *dsLacZ*-injected groups. n = total number of midguts examined.

We have recently shown that the HPx2/NOX5 system potentiates NO toxicity and mediates nitration of midgut epithelial cells in response to *Plasmodium* invasion. The potential participation of JNK signaling in the induction of these two enzymes and epithelial nitration was investigated. A robust increase in HPx2 and NOX5 expression was observed in the dsLacZ-injected control group (Figure 2A) in response to *Plasmodium* infection, as previously shown in uninjected females [8]; however, HPx2 was no longer induced in infected midguts and NOX5 expression was significantly reduced, relative to uninfected controls when JNK was silenced; and expression of both HPx2 and NOX5 is reduced in infected midguts when *jun* is silenced (Figure 2A, Table S4). The transcriptional induction of HPx2 in response to infection was more robust when *puc* was silenced, but NOX5 induction was no longer observed (Figure 2A, Table S4). In agreement with the overall transcriptional responses, when JNK was silenced, *in vivo* midgut nitration no longer increased in response to *Plasmodium* infection, was lower than in the uninfected controls when *jun* was silenced, while silencing *puc* had the opposite effect and enhanced the nitration response. (Figure 2B, Figure S3, Table S5).

Furthermore, co-silencing HPx2 (Figure 2C, Table S3) completely rescued the dramatic antiplasmodial effect of silencing *puc* alone. Co-silencing HPx2 increased the median number of oocysts/midgut from 0 to 17 (*p*<0.0001; KS test) and the prevalence of infection from 20% to 100% (*p*<0.0001; χ^2^ test). Co-silencing NOX5 and *puc* (Figure 2D, Table S3) increased the median number of oocysts/midgut from 0 to 10 (*p*<0.0001; KS test) to the same level as the dsLacZ control, and the prevalence of infection from 22% to 88% (*p*<0.0001; χ^2^ test). This indicates that HPx2 and NOX5 are downstream of *puc* and mediate, to a large extent, the antiplasmodial response triggered by the JNK activation.

### The JNK Pathway Regulates Expression of Hemocyte-Derived Antiplasmodial Effectors

*Jun* expression is induced in mosquito hemocytes 24 hpi with *P. berghei* [27], suggesting that JNK signaling in these cells could also be an important component of antiplasmodial immunity. TEP1 and FBN9 are proteins constitutively produced by hemocytes that are secreted into the mosquito hemolymph, bind to the surface of *P. berghei* ookinetes, and mediate parasite lysis [5], [6]. We investigated the hypothesis that these hemocyte-derived proteins are regulated by the JNK pathway and are important effectors of this signaling cascade.

**Figure 3:**
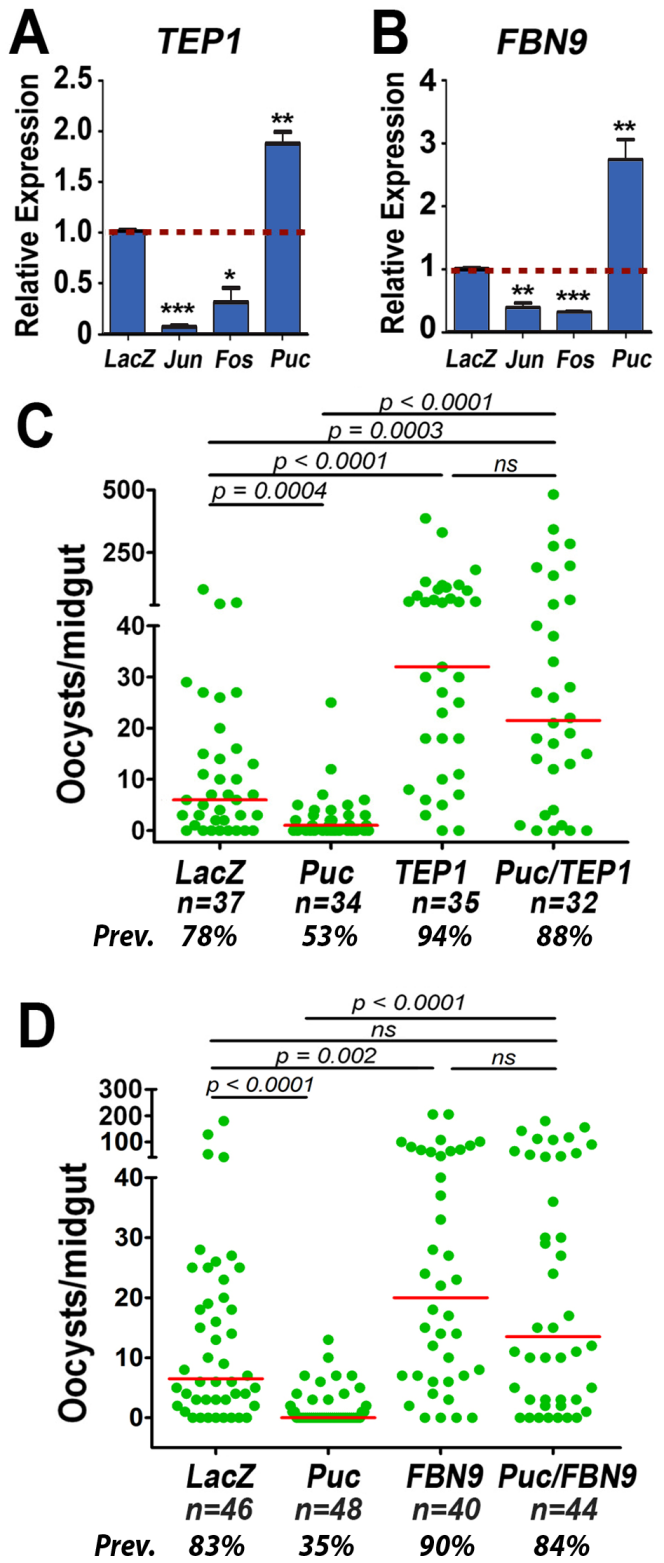
The JNK Pathway and Hemocyte Antiplasmodial Effector Genes. (A and B) Effect of silencing *jun*, *fos*, or *puc* on basal expression of *TEP1* (A) and *FBN9* (B) in circulating hemocytes. Mean expression level in silenced samples, relative to the dsLacZ-injected control that was adjusted to a value of “1” and is indicated by the red dotted line. The bars represent the SEM of two biological replicates from independent experiments (see Table S4). P-values determined by Student's-T test after log 2 transformation; *, p<0.05, **, p<0.01, ***, *p*<0.001. (C and D) Effect of co-silencing *TEP1* (C) or *FBN9* (D) on the phenotype of silencing negative the regulator *puc*. Green dots represent oocyst counts in individual midguts, and the horizontal red bar indicates median infection intensity. P-values were determined by Mann-Whitney test; ns, not significant. Graphs represent data from three biological replicates with comparable medians in the *dsLacZ*-injected groups (see Table S1) n = total number of midguts examined.

Silencing *jun* or *fos* significantly reduced TEP1 by 94% and 69%, respectively (*p*<0.001 and p<0.05; Student's t-test) and reduced FBN9 expression by 62% and 70%, respectively (Figure 3, A and B, Table S6) (*p*<0.01 and *p*<0.001; Student's t-test) but had no effect on the expression levels of other hemocyte-specific genes such as APL1A or APL1C, and *fos* silencing actually resulted in a modest increase in LRIM1 expression (Figure S4, Table S6). Conversely, silencing *puc* significantly increased expression of both TEP1 and FBN9 by 1.86 and 2.6 fold, respectively (Figure 3, A and B, Table S6) (p *p<0.01*; Student's t-test). Silencing JNK did not affect the total number of circulating hemocytes or the proportions of granulocytes, oenocytoids, or prohemocytes circulating in the mosquito (Figure S5). We have previously shown that induction of HPx2 and NOX5 mediates epithelial nitration and that the activity of these enzymes promotes both TEP1 binding to the ookinete surface and parasite lysis [8]. Participation of TEP1 and FBN9 as final effectors of the JNK antiplasmodial response was explored by co-silencing these genes with *puc*. Co-silencing TEP1 increased the median number of oocysts/midgut from 1 to 21.5 (*p*<0.0001; KS test) and the prevalence of infection from 53% to 88% (*p*<0.02, χ^2^ test) relative to silencing *puc* alone (Figure 3C, Table S3). Co-silencing FBN9 had a similar effect, increasing the median number of oocysts/midgut from 0 to 13.5 (p<0.0001; KS test) and the prevalence of infection from 35% to 84% (*p*<0.0001, χ^2^ test) (Figure 3D, Table S3).

### The JNK Pathway Contributes to Parasite Elimination in the *An. gambiae* Refractory Strain

The *An. gambiae* refractory (R) strain was selected to be refractory to *Plasmodium cynomolgi* (simian malaria) infection but also eliminates most *Plasmodium* species, including *P. berghei* [29]. In this mosquito strain, ookinetes develop and invade the midgut, but they are killed and covered with melanin, a black, insoluble pigment [29]. R females are in a chronic state of oxidative stress that is exacerbated by blood feeding [30], and TEP1 is known to be a critical mediator of *P. berghei* melanization and killing [5]. We have shown that the JNK pathway regulates expression of two enzymes that mediate midgut epithelial nitration: NOX5, an oxidase that generates ROS, and the heme peroxidase, HPX2. Furthermore, exposure of ookinetes to these nitration reactions as they traverse the midgut epithelial cell promotes TEP1 activation [8]. The hypothesis that the refractory phenotype may be mediated, at least in part, by the JNK signaling pathway was investigated.

**Figure 4:**
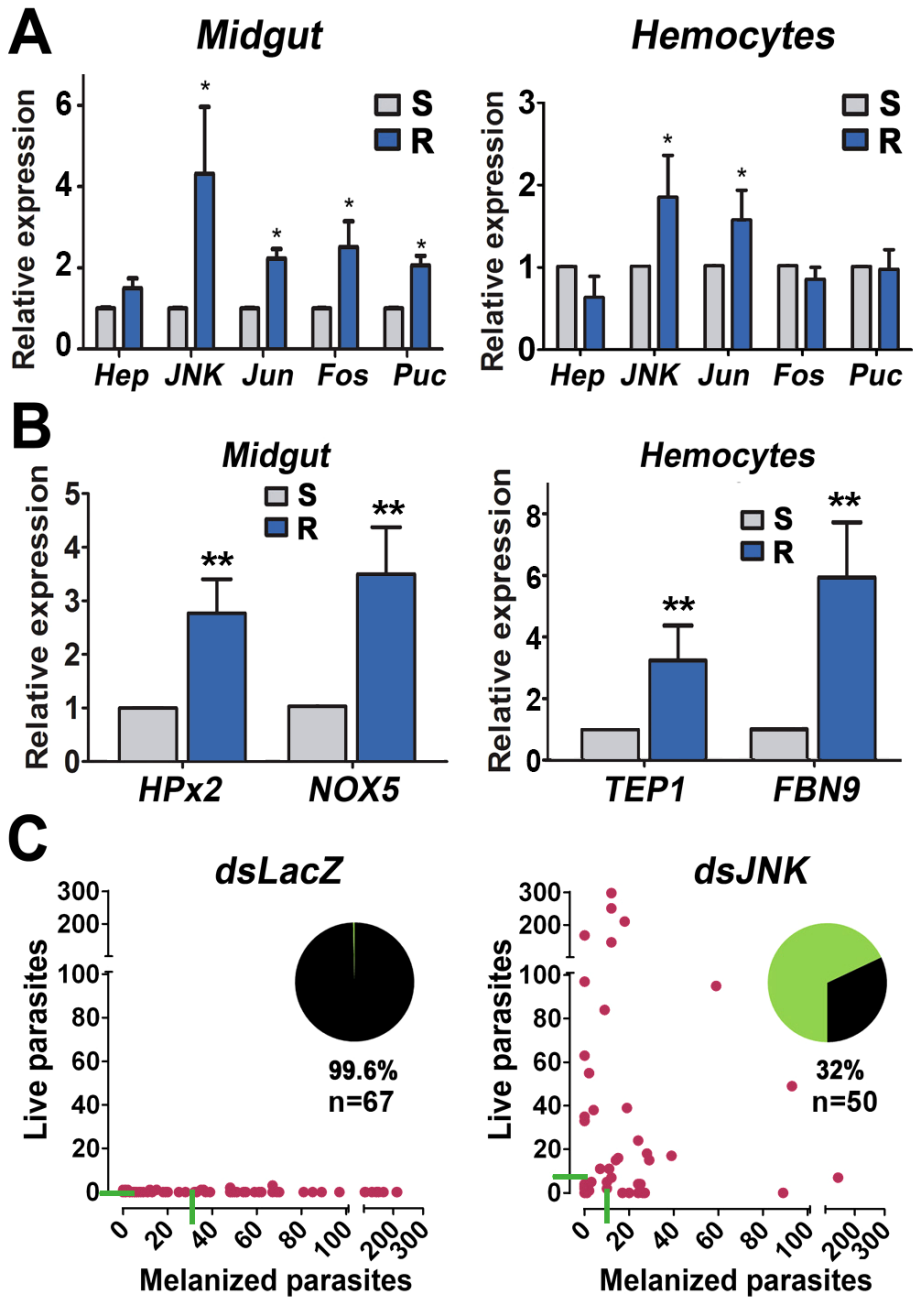
Participation of the JNK Pathway in L3–5 Mosquitoes Refractory (R) responses to *Plasmodium berghei* Infection. (A) Basal mRNA expression of genes from the JNK pathway in the midgut and hemocytes of G3 susceptible (S) (gray) and R (blue) mosquitoes. (B) Expression of effector genes regulated by the JNK pathway in S (gray) and R (blue) mosquitoes. Basal mRNA levels of HPx2 and NOX5 in the midgut, and of TEP1 and FBN9 in hemocytes. Graphs represent the expression level in R females, relative to S females, that were adjusted to a value of “1”; for R females samples the bars represent the SEM of three biological replicates from independent experiments (see Table S4). P-values determined by paired Student's-T test after log2 transformation; *, p<0.05, **, p<0.01, ***, *p*<0.001. (C) Effect of silencing JNK (right panel) in the number of melanized and live parasites on individual midguts of R mosquitoes. Red dots indicate the number of parasites on an individual midgut, live (y-axis) and melanized (x-axis). Green horizontal bars indicate median infection intensities. Inset pie graphs represent the percentage of total parasites for each group displaying a live (green) or melanized (black) phenotype; percentage displayed refers to melanized parasites. Graphs represent data from three biological replicates (see Table S10). (n = number of midguts analyzed).

We first compared the basal level of mRNA expression of the genes involved in JNK signaling between the susceptible (S) G3 *An. gambiae* and the R strain. The basal midgut expression of all the genes involved in JNK signaling was higher in the R strain. Midgut *jnk* expression was dramatically higher (4.3 fold), while the overexpression of *hep* was less prominent (1.5 fold) (Figure 4A, Table S7). The basal expression level of all genes of the JNK pathway (*hep*, *jnk*, *puc*, *jun*, and *fos*) was also significantly higher in whole body samples of R females, ranging from 2.1 to 4.8 fold (Figure S6, Table S7). Higher *puc* expression is indicative of increased JNK activation, because *puc* expression is transcriptionally regulated by the JNK pathway. In hemocytes, there was no difference in *hep*, *fos* and *puc* expression between the mosquito strains, but *jnk* and *jun* levels were also significantly higher in the R strain (Figure 4A, Table S7). Furthermore, expression of effector genes of the JNK pathway was also higher in the R strain. In the midgut, basal HPx2 and NOX5 expression was 2.8 and 3.5 fold times higher, respectively (Figure 4B, Table S8) (*p*<0.01; paired t-test for both); while in hemocytes, TEP1 and FBN9 expression was 3.2 and 5.9 fold higher in R mosquitoes, respectively (Figure 4B, Table S9) (*p*<0.01; paired t-test for both).

The contribution of the JNK pathway to the refractory phenotype was directly tested by reducing JNK expression via gene silencing. JNK silencing had a dramatic effect, increasing the prevalence of infection from 0 to 70% (Figure 4C) (p<0.0001; χ^2^ test), and the median number of oocysts from 0 to 6 oocysts/midgut (Figure 4C, Table S10) (*p*<0.001; KS test). The total number of parasites (live and melanized) was not significantly different between the dsLacZ control and the JNK-silenced group, indicating that a similar number of ookinetes invaded the midgut and that the difference in infection prevalence was due to ookinete survival once they traversed the midgut. Of the total number of parasites present, 99.6% of parasites were melanized in the dsLacZ group; this decreased to 32% when JNK was silenced (Figure 4C) (*p*<0.0001, χ^2^ test).

## Discussion

The immune response of *An. gambiae* mosquitoes against *Plasmodium* parasites is mediated by activation of immune-related signal transduction pathways. We carried out a functional characterization of five *An. gambiae* orthologs of genes known to mediate JNK signaling in *Drosophila*. Our studies implicate the JNK pathway as an important mediator of two coordinated steps of the mosquito anti-*Plasmodium* immune response and as a major determinant of the killing mechanism in a highly refractory strain of *An. gambiae*.

JNK signaling triggers the transcriptional activation of HPX2 and NOX5, two key enzymatic effectors of midgut epithelial cells, in response to ookinete invasion. Induction of these two enzymes potentiates nitration and limits *Plasmodium* survival. This was directly confirmed by the observation that midgut nitration is greatly diminished when JNK signaling is disrupted by silencing JNK or *jun*. Overactivation of the JNK pathway by silencing *puc*, greatly increased midgut HPx2 expression and nitration in response to *Plasmodium* infection. Interestingly, *puc* silencing did not induce higher levels of NOX5 expression. This enzyme generates reactive oxygen species that could be potentially toxic. Our results suggest that in the absence of *puc*, there might be alternative mechanisms that limit NOX5 expression,probably to prevent deleterious effects on the host. Previous studies in a variety of mammalian cell types have also shown that NOX5 and other NADPH oxidases are regulated by the JNK pathway [31]–[33] and induction of a nitrogen dioxide-producing heme peroxidase has also been shown to be mediated by the JNK pathway [34].

The process of nitration is clearly an essential step in the destruction of malaria parasites, evidenced by the significant increase in parasite survival upon silencing either HPx2 or NOX5. It is also a critical outcome of JNK activation, as the considerable resistance conferred by *puc* silencing is reverted by co-silencing either of these two enzymes (Figure 2). We therefore propose that the JNK pathway is part of an “alarm system” triggered by parasite invasion that activates expression of NOX5 and HPx2, two enzymes that catalyze nitration reactions, that label ookinetes for destruction as they traverse the mosquito midgut.

The dramatic reduction in TEP1 and FBN9 mRNA levels in hemocytes when the JNK pathway was disrupted by silencing *jun* or *fos* appears to be specific, because expression of other hemocyte-specific genes involved in the regulation of complement activation (APL1A, APL1C, and LRIM1) was not reduced. We also confirmed that the differences in expression were not due to significant changes in the number or type of hemocytes present in silenced mosquitoes. The co-silencing experiments with *puc* confirmed that both TEP1 and FBN9 are downstream of JNK. This indicates that the basal level of TEP1 and FBN9 expression in mosquito hemocytes is regulated by the JNK pathway and that both genes are important effectors of the lytic response mediated by this cascade.

Previous studies have shown that the R strain is in a chronic state of oxidative stress that is exacerbated when adult females take a blood meal [30]. Genome-wide transcriptional analysis revealed higher expression in the R strain of several immune genes, genes encoded by the mitochondrial genome, and genes involved in oxido/reductive processes or ROS detoxification relative to S females [30]. The R strain also exhibits impaired mitochondrial state-3 respiration and increased rate of electron leak [35]. NOX5 is a member of the NAPDH oxidase family and generates superoxide anion, which is quickly converted into hydrogen peroxide by superoxide dismutase (reviewed by Bedard and Kraus [36]). We found that the genes that mediate signaling (*hep*, *JNK*, *jun*, *fos*, and *puc*) and key downstream effectors of this pathway in the midgut (HPx2 and NOX5) and hemocytes (TEP1 and FBN9) have increased basal levels of expression. Higher levels of HPx2and NOX5 are expected to accelerate the rate of epithelial nitration, and higher hemolymph levels of TEP1 and FBN9 would promote parasite lysis. The increase in basal expression of NOX5 may be responsible, at least in part, for the higher constitutive levels of systemic ROS that have been observed in the R strain [30]. In *An. gambiae*, ROS levels have been shown to modulate immunity to both bacteria and *Plasmodium* [30], [37]. The dramatic reduction in melanization and the increase in parasite survival when JNK signaling is disrupted in the R strain confirm the key role of this pathway in mosquito antiplasmodial immunity.

We have recently shown that some *P. falciparum* strains, such as NF54, are able to infect the *An. gambiae* R strain and that silencing TEP1 did not enhance parasite survival, indicating that the mosquito complement-like system was not activated. In contrast, other parasite strains (such as 7G8) were almost completely eliminated through a TEP1-mediated mechanism [38]. Co-infection experiments with a *P. falciparum* strain that is melanized and one that survives suggest that survival is genetically determined by a parasite-autonomous mechanism, because the survival (or lack thereof) of one strain does not affect the outcome of the other strain in the same mosquito [38]. Together, this indicates that some *P. falciparum* strains are susceptible to a TEP1-dependent killing mechanism, while others have the capacity to evade it. Given the critical role of TEP1 as an effector of the JNK pathway, it is likely that *P. falciparum* strains also differ in their ability to avoid—or perhaps may even actively suppress—activation of this signaling cascade. Detailed studies on the participation of the JNK pathway in mosquito antiplasmodial responses to different *P. falciparum* strains are currently under way and may shed new insights into immune evasion strategies that promote human malaria transmission.

## Materials and Methods

### Ethics Statement

Public Health Service Animal Welfare Assurance #A4149-01 guidelines were followed according to the National Institutes of Health Animal (NIH) Office of Animal Care and Use (OACU). These studies were done according to the NIH animal study protocol (ASP) approved by the NIH Animal Care and User Committee (ACUC), with approval ID ASP-LMVR5.

### Mosquito Strains and Rearing

*An. gambiae* G3 and L3–5 mosquitoes were reared at 27°C with 80% humidity on a 12-h light/dark cycle. Cotton balls soaked in 10% sucrose in water were provided as previously described [39].

### Mosquito Tissue Dissections

Approximately 15–20 female mosquitoes 3- to 4 days post-emergence were removed cold anesthetized to immobilize them. Hemocytes from individual mosquitoes were extracted using the method outlined below, Trizol was added and samples were kept on ice. Then the head was severed from the thorax, and the thorax from the abdomen using a clean scalpel. The midgut and undeveloped ovaries were then pulled from the abdomen using fine forceps. Samples from each tissue were pooled together and stored in RNA*later* (Ambion, Austin, Texas, USA) in a microfuge tube. RNA was extracted, cDNA was generated, and gene expression was quantified using the methods indicated below for “Quantification of gene expression” (for tissues except hemocytes) or “Hemocyte collection and counting” (for hemocytes).

### Quantification of Gene Expression

Fifteen to twenty whole female mosquitoes or dissected tissues were homogenized in RNA*later* (Ambion) and subject to RNA extraction using RNA*easy* (Qiagen, Los Angeles, California, USA) kits according to the manufacturer's instructions and first-strand cDNA was synthesized using QuantiTect reverse transcriptase (Qiagen). Gene expression was assessed by SYBR green quantitative real-time PCR (DyNAmo HS; New England Biolabs, Beverly, Massachusetts, USA) using the CFX96 system (Bio-Rad, Hercules, California, USA). Each sample was assayed using two technical replicas and 2–3 biological replicates. The amount of cDNA template present in each sample was normalized using the expression *An. gambiae* ribosomal protein S7 as reference. Fold change values were derived using the 2^−ΔΔ^Ct method. The values were adjusted in each experiment by dividing each of the technical replicates in the control and treatment groups by the mean of the control group, thus adjusting the control groups to a value of “1”. The statistical analysis was done using the Student's T-test after log2 transformation of the mean value of each biological replicate for each treatment. Primers used are provided in Table S11; when appropriate, primers were verified against R strain sequences obtained by Solexa transcriptome sequencing of adult S and R females (Barillas-Mury Lab, unpublished). The primers used for TEP1 expression analysis in Figure 3 (S strain) are located in a polymorphic region between S and R strains of *A. gambiae*. For this reason, a different primer set in a conserved region was used for the TEP1 expression data presented in Figure 4 (comparison between S and R strains).

### *P. berghei* Maintenance and Infection

*P. berghei* (GFP-CON transgenic 259cl2 strain) parasites from frozen stocks were administered intraperitoneally to donor mice. When the parasitemias of donor mice reached 10–20%, 20–50 µl of infected blood was transferred to naïve mice via intraperitoneal injection. All mice were 3- to 5-week-old BALB/c females. Parasitemia was assessed by light microscopy inspection of Giemsa-stained thin smears obtained by tail snips. At 2–3 days post emergence, female mosquitoes were deprived of sucrose solution for 6–12 h, then allowed to feed on anesthetized mice infected with *P. berghei* at 3–7% parasitemia and exhibiting 1–3 exflagellation events per field, as previously described [40]. Where indicated, naïve blood-fed control mosquito groups were fed on uninfected mice of the same age. All *P. berghei*-infected mosquitoes and corresponding control mosquitoes were kept at 21°C and 80% humidity. Unless otherwise indicated, *P. berghei* infection intensities were quantified 7–9 days post infection (dpi) by epifluorescent microscopy inspection of dissected midguts containing GFP-expressing parasites fixed in 4% paraformaldehyde and mounted in Vectashield (Vector Labs, Burlingame, California, USA), enabling manual counting of fluorescent oocysts and/or melanized ookinetes.

### RNAi Gene-Silencing Assays

T7 promoter sequences were introduced at both ends using two different strategies. For LacZ, NOX5 and Tep1, cDNA fragments were amplified using the primers given in Table S11 and cloned into the pCRII-TOPO vector (Invitrogen, Carlsbad, California, USA) following the manufacturer's instructions. T7 promoters were introduced by amplifying the cloned insert using the primers: M13F: 5′-GTAAAACGA CGGCCAGT-3′ and M13R: 5′-CTCGAGTAATACGACTCACTATAGGGCAGGAAA CAGCTATGAC-3′, which anneal to the vector as previously reported [8]. These PCR products were used as templates for generating dsRNA as described below. For all other genes, the T7 sequences were included in the gene-specific primers and cDNA fragments of about ∼300-bp were generated (Table S11). For all genes, sense and antisense RNAs were synthesized simoultaneously from templates and purified using the T7 RNAi Megascript kit (Ambion), eluted in water, and concentrated to 3 µg/µl using a Microcon YM-100 filter (Millipore, Bedford, Massachusetts, USA). About 69 nl of this dsRNA preparation was injected into the thorax of cold-anesthetized, 2- to 3-day-old female mosquitoes using a nano-injector (Nanoject; Drummond Scientific, Broomall, Pennsylvania, USA) fitted with a glass capillary needle according to previous protocols. dsRNA targeting LacZ was used in each experiment to control for any unspecific effect of wounding and dsRNA exposure. Efficiency of silencing was quantified 2–3 days after dsRNA injection by real-time quantitative RT-PCR with the *An. gambiae* ribosomal S7 gene as the internal control for normalization. Primers for silencing verification are listed in Table S11, and silencing efficiencies are displayed in Figure S2.

### Immunoblotting

Midguts were dissected and cleaned of blood meal in cold, sterile PBS supplemented with 1% levamisole (Sigma-Aldrich, St. Louis, Missouri, USA). Pools of 5–10 midguts were transferred to a microfuge tube and homogenized in PBS with protease inhibitor, levamisole, and phosphoStop (Roche Applied Science, Madison, Wisconsin, USA), prepared using NuPAGE buffers and reducing agent (Invitrogen), and run on NuPAGE Bis-Tris 4–12% gels (Invitrogen) according to manufacturer's instructions. Proteins were then transferred from gels to membranes using the iBlot system (Invitrogen). Membranes were blocked in TBS with 5% milk +0 .05% Tween, washed, and incubated in fresh milk solution with primary antibody against JNK (1∶2000; Santa Cruz Biotechnology, Santa Cruz, California, USA) overnight. They were then washed and incubated in fresh milk solution with alkaline phosphatase-conjugated secondary antibody against rabbit (1∶5000) for 2 h with TBS washings between each step. Membranes were finally rinsed with TBS and incubated for 30 min (anti-JNK) with Western Blue substrate (Promega Corp., Madison, Wisconsin, USA) to visualize bands.

### *In vivo* Nitration Assays

Assays were performed according to previously established methods [8]. In brief, five midguts were dissected, fixed, and washed with PBS, then triturated and incubated in amino triazole (10 mg/ml). Pelleted midgut fragments were incubated with 2 mM levamisole, then blocked with PBT and washed. The pellet was subsequently resuspended in 50 µl of PBT, and five replicates of one-midgut equivalents (10 µl of the 50-µl suspension) were incubated overnight with anti-nitrotyrosine primary antibody diluted in PBT (1∶3,000) at 4°C. Samples were washed with PBT and 4 were incubated with a secondary alkaline phosphatase-conjugated antibody (1∶5,000) diluted in PBT, while the remaining sample was reserved as a background signal control. All samples were incubated with ρNPP–ρ-nitrophenylphosphate (Sigma Aldrich) and read in a spectrofluorometer plate reader at 405 nm. The relative nitration for each experimental treatment was confirmed in at least two independent experiments.

### Hemocyte Collection and Counting

Female mosquitoes were cold anesthetized and injected intrathoracically with a micropipette needle loaded with hemocyte perfusion buffer (60% Schneider's insect medium, 30% citrate, 10% FBS). After insertion of the needle into the thorax, a small incision was made in the lower abdomen, and buffer was dispensed through the needle and collected 2 µl at a time from the incision using siliconized pipet tips for a total of 10–12 µl. Perfusions were then either collected into a siliconized Eppendorf tube for RNA extraction or applied to a disposable hemocytometer (InCyto, Seoul, South Korea) for counting. For RNA extraction, tubes were centrifuged for 30 min at 12,000×*g* to pellet the cells; supernatant was removed, and 500 µl Trizol was added. RNA was isolated according to phenol/chloroform extraction as suggested by Trizol protocol. For counting, cells were visualized under light microscope with 40× objective. Cells contained within the marked grid were separated into three cell types (granulocyte, oenocytoid, prohemocyte) and counted accordingly. Population proportions were calculated and total numbers of cells per mosquito were determined by manufacturer's extrapolation.

### Statistical Analysis

Fold change differences in gene expression across groups were normalized by log transformation. The statistical analysis of differences in gene expression was done using the Student's T-test after log2 transformation of the mean value of each biological replicate from independent experiments. Oocyst distributions were determined not to be normal, and were compared to one another using the Kolmogorov-Smirnov (KS), Mann-Whitney tests and Kruskal-Wallis tests with Dunn's post-test (see Tables S3 and S10). When the median infection levels of the dsLacZ group of two or more biological replicates were not statistically different using the Mann-Whitney test, the data were merged (See Tables S3 and S10). Oocyst prevalences were compared using χ^2^ tests. Differences in nitration levels were compared using the Student's *t*-test. P-values represented in figures are given in corresponding figure legends and text. All statistical analyses were performed using Prism 5.01 software (GraphPad Software, La Jolla, California, USA).

## Supporting Information

Figure S1 **JNK protein midgut expression in response to** ***P. berghei*** **infection.** JNK was detected with commercial antibodies in Western Blots from midgut homogenates obtained from sugar-fed females (SF), control (C) females fed on a healthy mouse or infected females (I) fed on *P. berghei*-infected mouse. Samples were collected 24 and 48 h after feeding. The size of the reference molecular markers is expressed as kDa and is indicated by the dots on the left.
(DOCX)

Figure S2 **Silencing Efficiency in Sugar-Fed Mosquitoes.** Silencing efficiency in sugar-fed mosquitoes after systemic injection of dsRNA for the target gene relative to the expression level compared with dsLacZ-injected control mosquitoes. Whole body expression was determined in sugar fed females either 2 days (HPx2 and NOX5) or 3 days (all other genes) after injection. (Mean ± SE).
(DOCX)

Figure S3 **Effect of silencing** ***JNK*** **,** ***jun*** **or** ***puc*** **on infection-induced** ***in vivo*** **midgut nitration.** C, control mosquitoes fed on a healthy mouse (gray bars); I, infected mosquitoes fed on a *P. berghei*-infected mouse (blue bars). Graphs represent one of two biological replicates (see Figure 2B and Table S5); error bars indicate SEM of three technical replicates. P-value determined by Student's *t*-test; *, p<0.05, **, *p*<0.01.
(DOCX)

Figure S4 **Effect of Silencing Jun or Fos on LRIM1, APL1A and APL1C expression.** Hemocyte mRNA expression of LRIM1, APL1A, and APL1C genes was determined 3 days after systemic injection of either dsLacZ, dsJun or dsFos (Mean ± SEM). (* indicates *p*<0.05; Student's *t*-test)
(DOCX)

Figure S5 **Effect of Silencing JNK on hemocyte populations.** Effect of silencing JNK on the total number of hemocytes and the relative abundance of granulocytes, oenocytoids, and prohemocytes 4 days after systemic injection of dsLacZ or dsJNK (Mean ± SEM). No significant differences were observed (Student's *t*-test).
(DOCX)

Figure S6 **Relative expression of genes from the JNK pathway in susceptible and refractory** ***An. gambiae*** **mosquitoes.** Basal mRNA levels of *hep*, *JNK*, *jun* and *fos* in susceptible (S, gray) and refractory (R, blue) mosquitoes (Mean ± SEM). Graphs represent the expression level in R females, relative to S females, that were adjusted to a value of “1”; for R females samples the bars represent the SEM of three biological replicates (see Table S4). P-values determined by paired Student's-T test after log2 transformation; *, p<0.05, **, p<0.01,.
(DOCX)

Table S1 **Quantification of tissue-specific expression of JNK pathway members.**
(DOCX)

Table S2 **Time-course quantification of** ***P. berghei*** **-responsive expression of JNK pathway members.**
(DOCX)

Table S3 **Summary of oocyst data for all G3 infections.**
(DOCX)

Table S4 **Quantification of HPx2 and NOX5 expression in the midgut following silencing of JNK pathway members.**
(DOCX)

Table S5 **Quantification of nitration in the midgut following silencing of JNK pathway members.**
(DOCX)

Table S6 **Quantification of effector expression in hemocytes following silencing of JNK pathway members.**
(DOCX)

Table S7 **Quantification of JNK pathway member expression in G3 and L3–5 mosquitoes and tissues.**
(DOCX)

Table S8 **Quantification of HPx2 and NOX5 in G3 and L3–5 midguts.**
(DOCX)

Table S9 **Quantification of HPx2 and NOX5 in G3 and L3–5 hemocytes.**
(DOCX)

Table S10 **Summary of oocyst data for all L3–5 infections.**
(DOCX)

Table S11 **Primers used for dsRNA templates and silencing validation/real-time PCR.**
(DOCX)
