# Supplementary material for: The JNK Pathway Is a Key Mediator of Anopheles gambiae Antiplasmodial Immunity
Source: PLoS Pathog. 2013 Sep 5;9(9):e1003622. doi: 10.1371/journal.ppat.1003622 (PMC3764222; doi:10.1371/journal.ppat.1003622)
Supplement: Figure S1 — JNK protein midgut expression in response to P. berghei infection. JNK was detected with commercial antibodies in Western Blots from midgut homogenates obtained from sugar-fed females (SF), control (C) females fed on a healthy mouse or infected females (I) fed on P. berghei-infected mouse. Samples were collected 24 and 48 h after feeding. The size of the reference molecular markers is expressed as kDa and is indicated by the dots on the left. (DOCX) [file ppat.1003622.s001.docx]

**
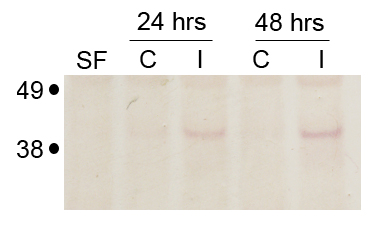
**

**Figure S1. JNK protein midgut expression in response to *P. berghei* infection.**  JNK was detected with commercial antibodies in Western Blots from midgut homogenates obtained from sugar-fed females (SF), control (C) females fed on a healthy mouse or infected females (I) fed on *P. berghei*-infected mouse. Samples were collected 24 and 48h after feeding. The size of the reference molecular markers is expressed as kDa and is indicated by the dots on the left.
