# Supplementary material for: The JNK Pathway Is a Key Mediator of Anopheles gambiae Antiplasmodial Immunity
Source: PLoS Pathog. 2013 Sep 5;9(9):e1003622. doi: 10.1371/journal.ppat.1003622 (PMC3764222; doi:10.1371/journal.ppat.1003622)
Supplement: Figure S2 — Silencing Efficiency in Sugar-Fed Mosquitoes. Silencing efficiency in sugar-fed mosquitoes after systemic injection of dsRNA for the target gene relative to the expression level compared with dsLacZ-injected control mosquitoes. Whole body expression was determined in sugar fed females either 2 days (HPx2 and NOX5) or 3 days (all other genes) after injection. (Mean ± SE). (DOCX) [file ppat.1003622.s002.docx]

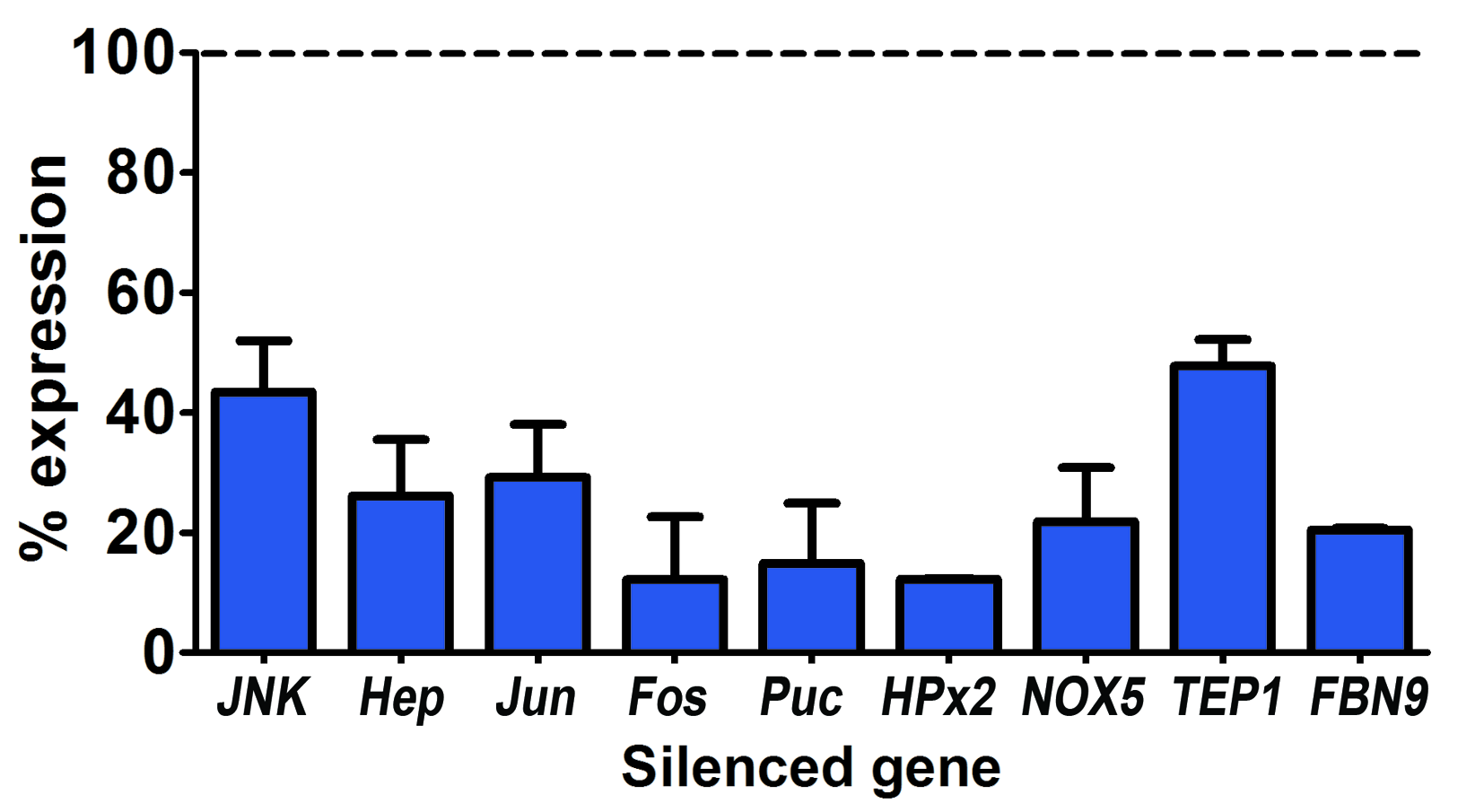


F**igure S2. Silencing Efficiency in Sugar-Fed Mosquitoes.** Silencing efficiency in sugar-fed mosquitoes after systemic injection of dsRNA for the target gene relative to the expression level compared with dsLacZ-injected control mosquitoes. Whole body expression was determined in sugar fed females either 2 days (HPx2 and NOX5) or 3 days (all other genes) after injection. (Mean ± SEM).
