# Supplementary material for: The JNK Pathway Is a Key Mediator of Anopheles gambiae Antiplasmodial Immunity
Source: PLoS Pathog. 2013 Sep 5;9(9):e1003622. doi: 10.1371/journal.ppat.1003622 (PMC3764222; doi:10.1371/journal.ppat.1003622)
Supplement: Figure S3 — Effect of silencing JNK , jun or puc on infection-induced in vivo midgut nitration. C, control mosquitoes fed on a healthy mouse (gray bars); I, infected mosquitoes fed on a P. berghei-infected mouse (blue bars). Graphs represent one of two biological replicates (see Figure 2B and Table S5); error bars indicate SEM of three technical replicates. P-value determined by Student's t-test; *, p<0.05, **, p<0.01. (DOCX) [file ppat.1003622.s003.docx]

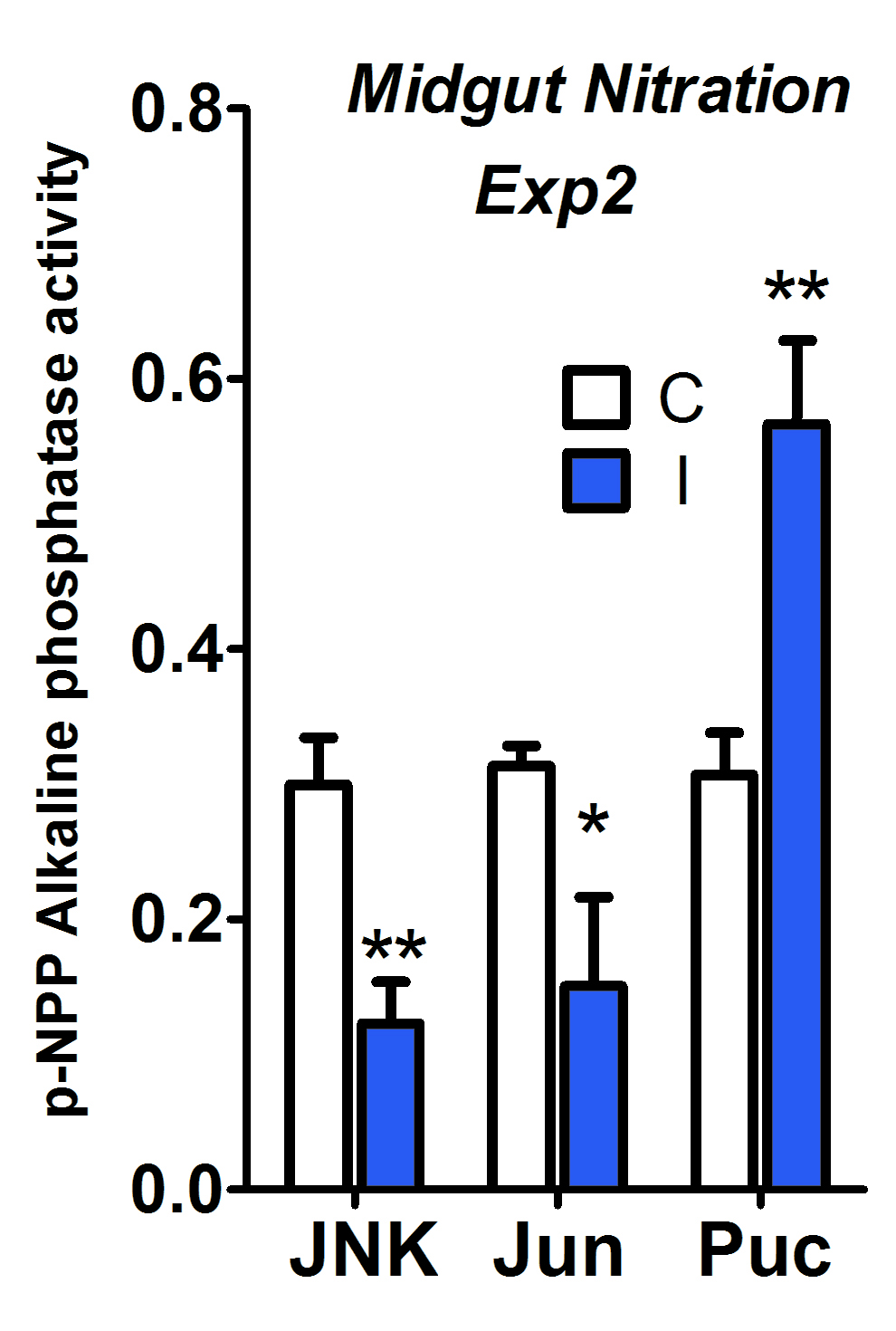


**Figure S3**. **Effect of silencing *JNK*, *jun* or *puc* on infection-induced *in vivo* midgut nitration.** C, control mosquitoes fed on a healthy mouse (gray bars); I, infected mosquitoes fed on a *P. berghei*-infected mouse (blue bars). Graphs represent one of two biological replicates (see Figure 2B and Table S5); error bars indicate SEM of three technical replicates. P-value determined by Student’s *t*‑test; *, p<0.05; **, *p* < 0.01.
