# Supplementary material for: The JNK Pathway Is a Key Mediator of Anopheles gambiae Antiplasmodial Immunity
Source: PLoS Pathog. 2013 Sep 5;9(9):e1003622. doi: 10.1371/journal.ppat.1003622 (PMC3764222; doi:10.1371/journal.ppat.1003622)
Supplement: Figure S4 — Effect of Silencing Jun or Fos on LRIM1, APL1A and APL1C expression. Hemocyte mRNA expression of LRIM1, APL1A, and APL1C genes was determined 3 days after systemic injection of either dsLacZ, dsJun or dsFos (Mean ± SEM). (* indicates p<0.05; Student's t-test) (DOCX) [file ppat.1003622.s004.docx]

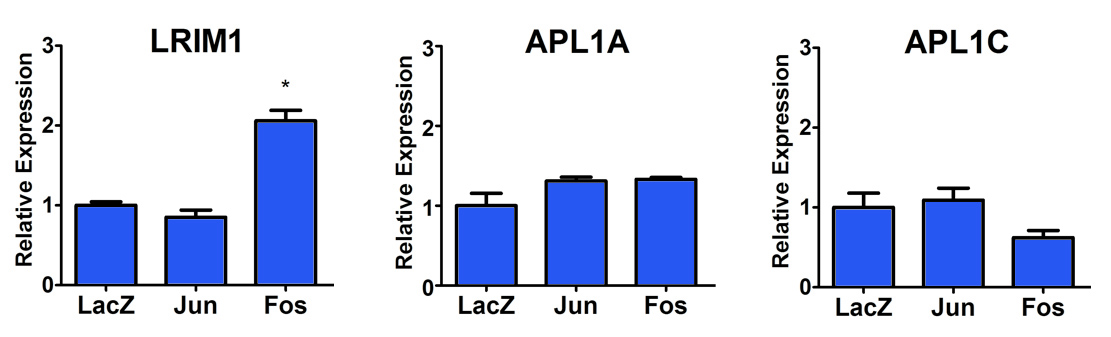


**Figure S4. Effect of Silencing Jun or Fos on LRIM1, APL1A and APL1C expression**. Hemocyte mRNA expression of LRIM1, APL1A, and APL1C genes was determined 3 days after systemic injection of either dsLacZ, dsJun or dsFos (Mean ± SEM). (* indicates *p* < 0.05; Student’s *t*‑test)
