# Supplementary material for: The JNK Pathway Is a Key Mediator of Anopheles gambiae Antiplasmodial Immunity
Source: PLoS Pathog. 2013 Sep 5;9(9):e1003622. doi: 10.1371/journal.ppat.1003622 (PMC3764222; doi:10.1371/journal.ppat.1003622)
Supplement: Figure S5 — Effect of Silencing JNK on hemocyte populations. Effect of silencing JNK on the total number of hemocytes and the relative abundance of granulocytes, oenocytoids, and prohemocytes 4 days after systemic injection of dsLacZ or dsJNK (Mean ± SEM). No significant differences were observed (Student's t-test). (DOCX) [file ppat.1003622.s005.docx]

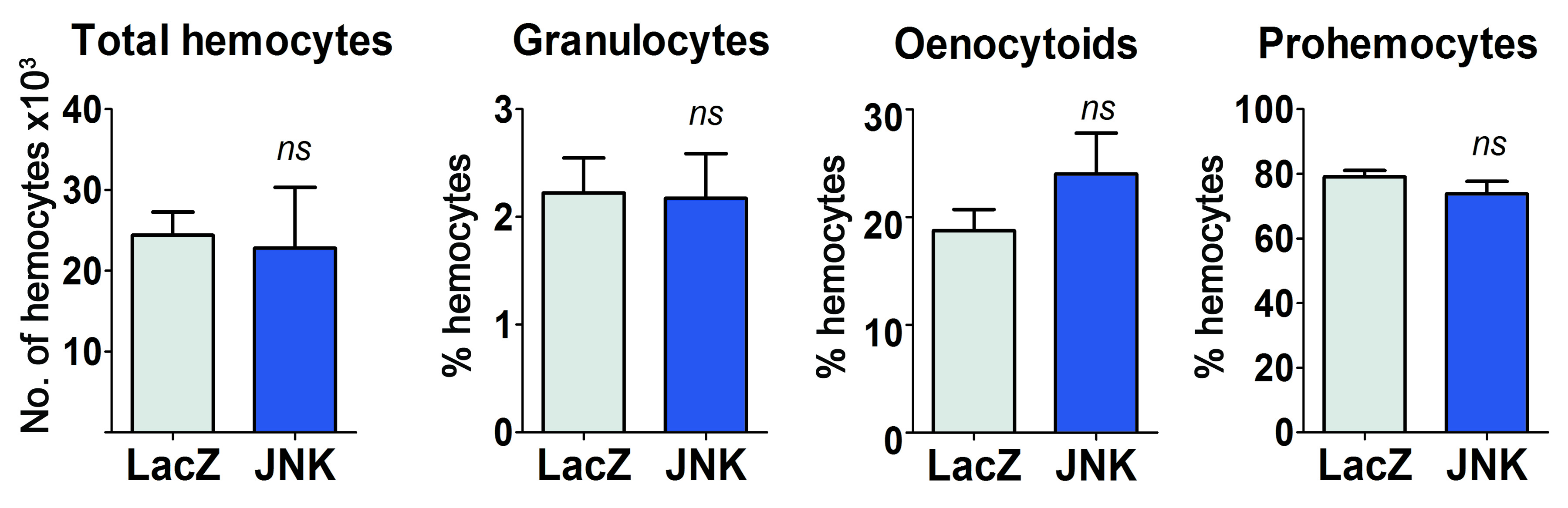


**Figure S5. Effect of Silencing JNK on hemocyte populations.** Effect of silencing JNK on the total number of hemocytes and the relative abundance of granulocytes, oenocytoids, and prohemocytes 4 days after systemic injection of dsLacZ or dsJNK (Mean ± SEM). No significant differences were observed (Student’s *t*‑test).
