# Supplementary material for: The JNK Pathway Is a Key Mediator of Anopheles gambiae Antiplasmodial Immunity
Source: PLoS Pathog. 2013 Sep 5;9(9):e1003622. doi: 10.1371/journal.ppat.1003622 (PMC3764222; doi:10.1371/journal.ppat.1003622)
Supplement: Figure S6 — Relative expression of genes from the JNK pathway in susceptible and refractory An. gambiae mosquitoes. Basal mRNA levels of hep, JNK, jun and fos in susceptible (S, gray) and refractory (R, blue) mosquitoes (Mean ± SEM). Graphs represent the expression level in R females, relative to S females, that were adjusted to a value of “1”; for R females samples the bars represent the SEM of three biological replicates (see Table S4). P-values determined by paired Student's-T test after log2 transformation; *, p<0.05, **, p<0.01,. (DOCX) [file ppat.1003622.s006.docx]

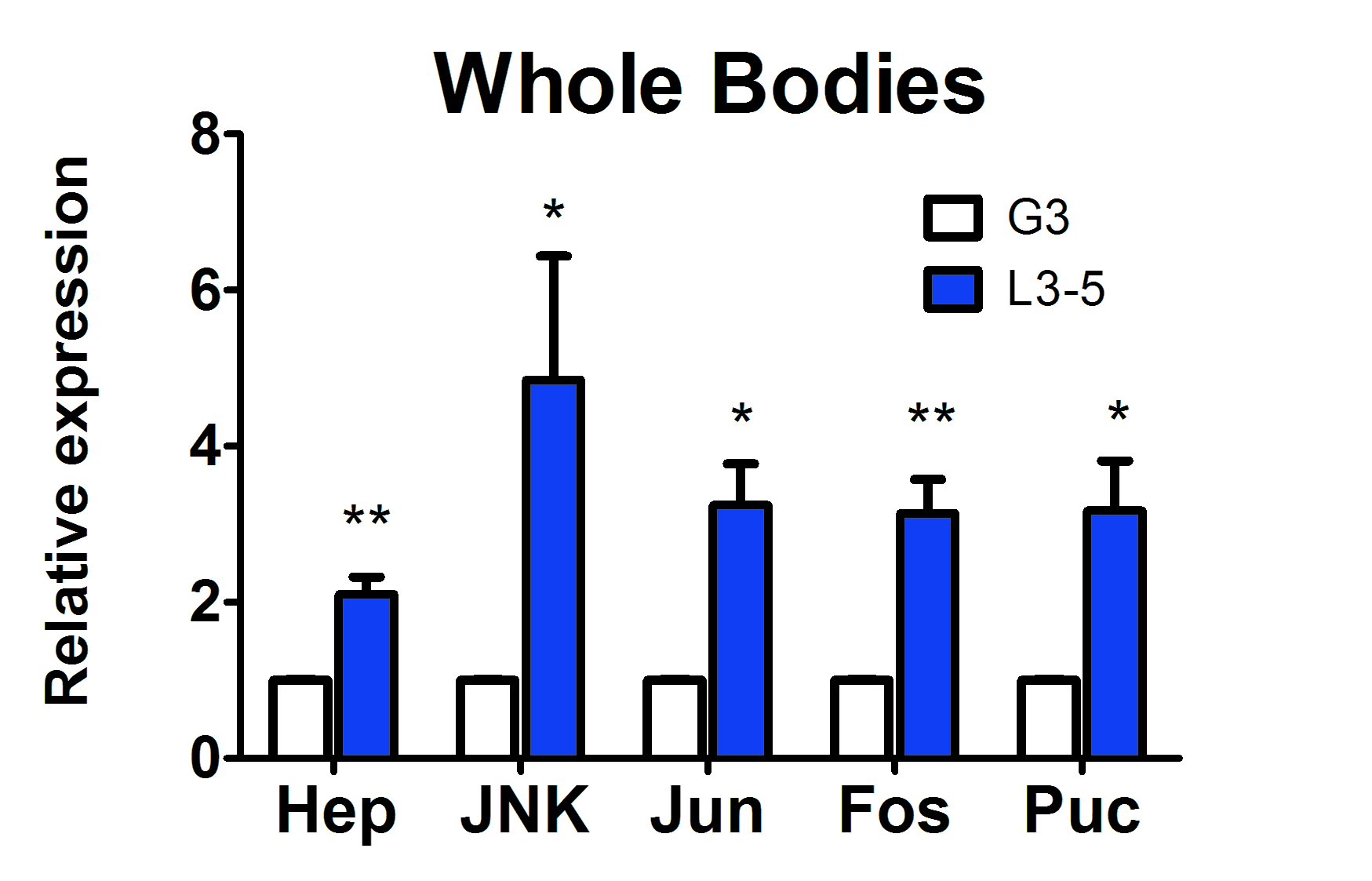


**Figure S6. Relative expression of genes from the JNK pathway in susceptible and refractory *An. gambiae* mosquitoes.** Basal mRNA levels of *hep, JNK, jun* and *fos* in susceptible (S, gray) and refractory (R, blue) mosquitoes. Graphs represent the expression level in R females, relative to S females, that were adjusted to a value of “1”; for R females samples the bars represent the SEM of three biological replicates (see Table S4). P-values determined by paired Student’s-T test after log2 transformation; *, p<0.05, **, p<0.01.
