# Supplementary material for: The JNK Pathway Is a Key Mediator of Anopheles gambiae Antiplasmodial Immunity
Source: PLoS Pathog. 2013 Sep 5;9(9):e1003622. doi: 10.1371/journal.ppat.1003622 (PMC3764222; doi:10.1371/journal.ppat.1003622)
Supplement: Table S1 — Quantification of tissue-specific expression of JNK pathway members. (DOCX) [file ppat.1003622.s007.docx]

**Table S1: Quantification of tissue-specific expression of JNK pathway members**

| Sample | Hep | | JNK | | Jun | | Fos | | Puc | |
| --- | --- | --- | --- | --- | --- | --- | --- | --- | --- | --- |
|  | *Exp1* | *Exp2* | *Exp1* | *Exp2* | *Exp1* | *Exp2* | *Exp1* | *Exp2* | *Exp1* | *Exp2* |
| Midgut | 1.00 | 1.00 | 1.00 | 1.00 | 1.00 | 1.00 | 1.00 | 1.00 | 1.00 | 1.00 |
| Head | ND | 0.06 | ND | ND | 0.15 | 0.15 | ND | ND | ND | 0.01 |
| Thorax | 15.77 | 8.41 | 5.18 | 3.08 | 1.76 | 1.84 | 3.26 | 2.17 | 0.95 | 1.34 |
| Abdomen | 2.50 | 1.87 | 2.41 | 2.82 | 1.00 | 1.42 | 2.11 | 2.15 | 0.53 | 0.83 |
| Hemocytes | 3.81 | 2.38 | 0.63 | 0.76 | 0.74 | 0.84 | 1.30 | 1.02 | 0.33 | 0.69 |
| Ovary | 1.09 | 1.15 | 3.17 | 6.22 | 0.50 | 1.58 | 1.51 | 2.60 | 0.11 | 0.44 |

Exp, experiment; ND, not detectable
