# Supplementary material for: The JNK Pathway Is a Key Mediator of Anopheles gambiae Antiplasmodial Immunity
Source: PLoS Pathog. 2013 Sep 5;9(9):e1003622. doi: 10.1371/journal.ppat.1003622 (PMC3764222; doi:10.1371/journal.ppat.1003622)
Supplement: Table S2 — Time-course quantification of P. berghei -responsive expression of JNK pathway members. (DOCX) [file ppat.1003622.s008.docx]

**Table S2: Time-course quantification of *P. berghei*-responsive expression of JNK pathway members**

| Time | Fed | Hep | | | JNK | | | Jun | | | Fos | | | Puc | | |
| --- | --- | --- | --- | --- | --- | --- | --- | --- | --- | --- | --- | --- | --- | --- | --- | --- |
| (hpf) |  | *Exp1* | *Exp2* | *Exp3* | *Exp1* | *Exp2* | *Exp3* | *Exp1* | *Exp2* | *Exp3* | *Exp1* | *Exp2* | *Exp3* | *Exp1* | *Exp2* | *Exp3* |
| 0 | Sugar | 1.00 | 1.00 | 1.00 | 1.00 | 1.00 | 1.00 | 1.00 | 1.00 | 1.00 | 1.00 | 1.00 | 1.00 | 1.00 | 1.00 | 1.00 |
| 12 | C | 0.94 | 1.14 | 0.35 | 7.97 | 2.05 | 0.34 | 1.59 | 0.59 | 0.57 | 1.73 | 0.89 | 0.14 | 3.22 | 0.44 | 0.67 |
| 12 | I | 1.07 | 0.50 | 0.77 | 9.09 | 1.29 | 2.04 | 1.56 | 0.41 | 0.54 | 4.03 | 0.51 | 0.88 | 3.72 | 0.24 | 0.44 |
| 24 | C | 1.35 | 0.83 | 2.55 | 9.05 | 1.48 | 2.57 | 2.06 | 0.55 | 1.72 | 2.67 | 0.64 | 1.18 | 5.04 | 0.41 | 1.14 |
| 24 | I | 1.81 | 0.64 | 0.95 | 12.11 | 1.69 | 3.46 | 9.67 | 2.27 | 3.07 | 4.45 | 1.28 | 1.23 | 18.84 | 0.63 | 1.15 |
| 48 | C | 2.21 | 1.97 | 1.36 | 7.19 | 2.62 | 1.67 | 6.27 | 2.38 | 1.31 | 3.79 | 1.74 | 1.51 | 15.77 | 1.95 | 0.76 |
| 48 | I | 1.86 | 2.14 | 2.05 | 36.72 | 3.70 | 4.63 | 8.11 | 2.93 | 4.13 | 4.33 | 3.58 | 2.76 | 15.65 | 3.62 | 2.82 |

Exp, experiment; hpf, hours post infection; C, control blood-fed; I, infected blood-fed
