# Supplementary material for: The JNK Pathway Is a Key Mediator of Anopheles gambiae Antiplasmodial Immunity
Source: PLoS Pathog. 2013 Sep 5;9(9):e1003622. doi: 10.1371/journal.ppat.1003622 (PMC3764222; doi:10.1371/journal.ppat.1003622)
Supplement: Table S3 — Summary of oocyst data for all G3 infections. (DOCX) [file ppat.1003622.s009.docx]

**Table S3: Summary of oocyst data for all G3 infections**

| Figure | RNAi | *Exp 1* | | *Exp 2* | | *Exp 3* | | Combined | | | | | | |
| --- | --- | --- | --- | --- | --- | --- | --- | --- | --- | --- | --- | --- | --- | --- |
|  |  | N | Med | N | Med | N | Med | N | Med | Prev (%) | M-W (to LacZ) | KS | K-W | Dunn’s (to LacZ) |
| 1D | LacZ | 17 | 2 | 25 | 4 | 15 | 6 | 57 | 5 | 68.4 |  | <.0001 | <.0001 |  |
|  | JNK | 18 | 28 | 20 | 14.5 | 13 | 25 | 54 | 24.5 | 85.2 | .0003 | <.0001 | <.0001 | ** |
|  | Fos | 21 | 38 | 22 | 21.5 | 19 | 12 | 59 | 21 | 76.3 | .0078 | <.0001 | <.0001 | * |
|  | Puc | 20 | 0 | 17 | 3 | 22 | 0 | 59 | 0 | 40.7 | <.0001 | <.0001 | <.0001 | ** |
| 1D | LacZ | 21 | 2 | 17 | 8 | 16 | 5 | 54 | 5 | 70.4 |  | <.0001 | n/a | n/a |
|  | Hep | 21 | 22 | 17 | 188 | 11 | 18 | 49 | 23 | 73.5 | .0027 | <.0001 | n/a | n/a |
| 1E | LacZ | 13 | 4 | 18 | 14 | 21 | 7 | 53 | 8 | 83.0 |  | <.0001 | <.0001 |  |
|  | Puc | 12 | 0 | 15 | 0 | 21 | 0 | 48 | 0 | 33.3 | <.0001 | <.0001 | <.0001 | *** |
|  | Jun | 11 | 111 | 15 | 19 | 17 | 17 | 43 | 31 | 86.0 | .0132 | .0002 | <.0001 | * |
|  | Puc/Jun | 14 | 76 | 15 | 5 | 16 | 5 | 45 | 10 | 84.4 | .4010 | <.0001 | <.0001 | ns |
| 2C | LacZ | 9 | 14 | 10 | 4 | 16 | 8 | 35 | 7 | 80.0 |  | .0004 | <.0001 |  |
|  | Puc | 10 | 0 | 14 | 0 | 22 | 0 | 46 | 0 | 19.6 | <.0001 | <.0001 | <.0001 | *** |
|  | HPx2 | 9 | 19 | 7 | 14 | 14 | 27 | 30 | 22 | 93.3 | .0055 | .0202 | <.0001 | * |
|  | Puc/HPx2 | 8 | 25 | 6 | 13 | 14 | 17 | 28 | 17 | 100.0 | .0346 | <.0001 | <.0001 | ns |
| 2D | LacZ | 10 | 16 | 17 | 5 | 16 | 8.5 | 43 | 7 | 81.4 |  | <.0001 | <.0001 |  |
|  | Puc | 10 | 0 | 22 | 0 | 22 | 0 | 54 | 0 | 22.2 | <.0001 | <.0001 | <.0001 | *** |
|  | NOX5 | 10 | 40.5 | 14 | 15 | 15 | 21 | 39 | 18 | 94.9 | .0020 | .0018 | <.0001 | * |
|  | Puc/NOX5 | 11 | 15.5 | 16 | 10 | 22 | 6 | 49 | 10 | 87.8 | .6576 | <.0001 | <.0001 | ns |
| 3C | LacZ | 10 | 5.5 | 18 | 8.5 | 9 | 3 | 37 | 6 | 78.4 |  | <.0001 | <.0001 |  |
|  | Puc | 10 | 0 | 14 | 1 | 9 | 1.5 | 34 | 1 | 52.9 | .0004 | <.0001 | <.0001 | * |
|  | TEP1 | 10 | 31 | 17 | 25 | 8 | 51 | 35 | 32 | 94.3 | <.0001 | .0002 | <.0001 | *** |
|  | Puc/TEP1 | 10 | 34 | 13 | 27 | 9 | 15 | 32 | 21.5 | 87.5 | .0029 | <.0001 | <.0001 | * |
| 3D | LacZ | 18 | 10.5 | 12 | 8 | 16 | 5 | 46 | 6.5 | 82.6 |  | <.0001 | <.0001 |  |
|  | Puc | 17 | 0 | 12 | 0 | 19 | 0 | 48 | 0 | 35.4 | <.0001 | <.0001 | <.0001 | *** |
|  | FBN9 | 14 | 25.5 | 11 | 15 | 15 | 18 | 40 | 20 | 90.0 | .0020 | <.0001 | <.0001 | ** |
|  | Puc/FBN9 | 14 | 57 | 11 | 27 | 19 | 5 | 44 | 13.5 | 84.1 | .2884 | <.0001 | <.0001 | ns |

Exp, experiment; Med, median; Prev, prevalence; M-W, Mann-Whitney; KS, Kolmogorov-Smirnov; K-W, Kruskal-Wallis; ns, not significant.
