# Supplementary material for: The JNK Pathway Is a Key Mediator of Anopheles gambiae Antiplasmodial Immunity
Source: PLoS Pathog. 2013 Sep 5;9(9):e1003622. doi: 10.1371/journal.ppat.1003622 (PMC3764222; doi:10.1371/journal.ppat.1003622)
Supplement: Table S4 — Quantification of HPx2 and NOX5 expression in the midgut following silencing of JNK pathway members. (DOCX) [file ppat.1003622.s010.docx]

**Table S4: Quantification of HPx2 and NOX5 expression in the midgut following silencing of JNK pathway members**

| RNAi | Feeding | HPx2 | | | NOX5 | | |
| --- | --- | --- | --- | --- | --- | --- | --- |
|  |  | *Exp1* | *Exp2* | *Exp3* | *Exp1* | *Exp2* | *Exp3* |
| LacZ | control | 1.00 | 1.00 | 1.00 | 1.00 | 1.00 | 1.00 |
|  | infected | 1.44 | 1.69 | 1.77 | 2.84 | 2.09 | 4.07 |
| JNK | control | 1.00 | 1.00 | 1.00 | 1.00 | 1.00 | 1.00 |
|  | infected | 1.07 | 0.50 | 0.98 | 0.29 | 0.86 | 0.13 |
| Jun | control | 1.00 | 1.00 | 1.00 | 1.00 | 1.00 | 1.00 |
|  | infected | 0.52 | 0.43 | 0.61 | 0.37 | 0.25 | 0.65 |
| Puc | control | 1.00 | 1.00 | 1.00 | 1.00 | 1.00 | 1.00 |
|  | infected | 3.39 | 2.52 | 2.90 | 1.38 | 0.39 | 1.15 |

Exp, experiment
