# Supplementary material for: The JNK Pathway Is a Key Mediator of Anopheles gambiae Antiplasmodial Immunity
Source: PLoS Pathog. 2013 Sep 5;9(9):e1003622. doi: 10.1371/journal.ppat.1003622 (PMC3764222; doi:10.1371/journal.ppat.1003622)
Supplement: Table S5 — Quantification of nitration in the midgut following silencing of JNK pathway members. (DOCX) [file ppat.1003622.s011.docx]

**Table S5: Quantification of nitration in the midgut following silencing of JNK pathway members**

| RNAi | Feeding | Absorbance Units | |
| --- | --- | --- | --- |
|  |  | *Exp1* | *Exp2* |
| LacZ | control | .320 | .318 |
|  | infected | .524 | .350 |
| JNK | control | .258 | .300 |
|  | infected | .218 | .123 |
| Jun | control | .455 | .314 |
|  | infected | .044 | .052 |
| Puc | control | .301 | .307 |
|  | infected | .886 | .567 |

Exp, experiment
