# Supplementary material for: The JNK Pathway Is a Key Mediator of Anopheles gambiae Antiplasmodial Immunity
Source: PLoS Pathog. 2013 Sep 5;9(9):e1003622. doi: 10.1371/journal.ppat.1003622 (PMC3764222; doi:10.1371/journal.ppat.1003622)
Supplement: Table S6 — Quantification of effector expression in hemocytes following silencing of JNK pathway members. (DOCX) [file ppat.1003622.s012.docx]

T**able S6: Quantification of effector expression in hemocytes following silencing of JNK pathway members**

| RNAi | TEP1 | | FBN9 | | LRIM1 | | APL1A | | APL1C | |
| --- | --- | --- | --- | --- | --- | --- | --- | --- | --- | --- |
|  | *Exp1* | *Exp2* | *Exp1* | *Exp2* | *Exp1* | *Exp2* | *Exp1* | *Exp2* | *Exp1* | *Exp2* |
| LacZ | 1.00 | 1.00 | 1.00 | 1.00 | 1.00 | 1.00 | 1.00 | 1.00 | 1.00 | 1.00 |
| Jun | .042 | .083 | 0.25 | 0.24 | 0.85 | 0.35 | 1.34 | 1.31 | 1.09 | 1.84 |
| Fos | .059 | .552 | 0.31 | 0.31 | 2.06 | 1.72 | 1.60 | 1.33 | 0.62 | 0.62 |
| Puc | 2.11 | 1.76 | 3.31 | 2.89 |  |  |  |  |  |  |

Exp, experiment
