# Supplementary material for: The JNK Pathway Is a Key Mediator of Anopheles gambiae Antiplasmodial Immunity
Source: PLoS Pathog. 2013 Sep 5;9(9):e1003622. doi: 10.1371/journal.ppat.1003622 (PMC3764222; doi:10.1371/journal.ppat.1003622)
Supplement: Table S7 — Quantification of JNK pathway member expression in G3 and L3–5 mosquitoes and tissues. (DOCX) [file ppat.1003622.s013.docx]

**Table S7: Quantification of JNK pathway member expression in G3 and L3-5 mosquitoes and tissues**

| Sample* | Hep | | | JNK | | | Jun | | | Fos | | | Puc | | |
| --- | --- | --- | --- | --- | --- | --- | --- | --- | --- | --- | --- | --- | --- | --- | --- |
|  | *Exp1* | *Exp2* | *Exp3* | *Exp1* | *Exp2* | *Exp3* | *Exp1* | *Exp2* | *Exp3* | *Exp1* | *Exp2* | *Exp3* | *Exp1* | *Exp2* | *Exp3* |
| G3 WB | 1.00 | 1.00 | 1.00 | 1.00 | 1.00 | 1.00 | 1.00 | 1.00 | 1.00 | 1.00 | 1.00 | 1.00 | 1.00 | 1.00 | 1.00 |
| L3-5 WB | 2.55 | 1.89 | 1.85 | 4.57 | 2.24 | 7.73 | 4.30 | 2.59 | 2.83 | 3.95 | 2.44 | 3.02 | 3.87 | 3.75 | 1.89 |
| G3 Mg | 1.00 | 1.00 | 1.00 | 1.00 | 1.00 | 1.00 | 1.00 | 1.00 | 1.00 | 1.00 | 1.00 | 1.00 | 1.00 | 1.00 | 1.00 |
| L3-5 Mg | 0.73 | 1.85 | 1.90 | 3.20 | 10.83 | 2.19 | 2.40 | 2.76 | 1.53 | 1.46 | 4.44 | 2.81 | 1.43 | 2.74 | 2.01 |
| G3 Hc | 1.00 | 1.00 | 1.00 | 1.00 | 1.00 | 1.00 | 1.00 | 1.00 | 1.00 | 1.00 | 1.00 | 1.00 | 1.00 | 1.00 | 1.00 |
| L3-5 Hc | 0.79 | 0.41 | 0.88 | 1.22 | 2.14 | 2.12 | 1.96 | 1.53 | 1.50 | 0.83 | 0.85 | 0.82 | 0.78 | 1.25 | 0.86 |

Exp, experiment; WB, whole body; Mg, midgut; Hc, hemocytes

*Mosquitoes used for whole, midgut and hemocyte assessment were from independent mosquito groups; a total of 9 G3-L3-5 age matched cohorts are represented in this table (3 biological replicates per strain per sample type).
