# Supplementary material for: The JNK Pathway Is a Key Mediator of Anopheles gambiae Antiplasmodial Immunity
Source: PLoS Pathog. 2013 Sep 5;9(9):e1003622. doi: 10.1371/journal.ppat.1003622 (PMC3764222; doi:10.1371/journal.ppat.1003622)
Supplement: Table S8 — Quantification of HPx2 and NOX5 in G3 and L3–5 midguts. (DOCX) [file ppat.1003622.s014.docx]

**Table S8: Quantification of HPx2 and NOX5 in G3 and L3-5 midguts**

| Sample | HPx2 | | | NOX5 | | |
| --- | --- | --- | --- | --- | --- | --- |
|  | *Exp1* | *Exp2* | *Exp3* | *Exp1* | *Exp2* | *Exp3* |
| G3 Midgut | 1.00 | 1.00 | 1.00 | 1.00 | 1.00 | 1.00 |
| L3-5 Midgut | 3.55 | 2.54 | 2.21 | 3.95 | 4.23 | 2.56 |

Exp, experiment
