# Supplementary material for: The JNK Pathway Is a Key Mediator of Anopheles gambiae Antiplasmodial Immunity
Source: PLoS Pathog. 2013 Sep 5;9(9):e1003622. doi: 10.1371/journal.ppat.1003622 (PMC3764222; doi:10.1371/journal.ppat.1003622)
Supplement: Table S9 — Quantification of HPx2 and NOX5 in G3 and L3–5 hemocytes. (DOCX) [file ppat.1003622.s015.docx]

**Table S9: Quantification of TEP1 and FBN9 in G3 and L3-5 hemocytes**

| Sample | TEP1 | | | FBN9 | | |
| --- | --- | --- | --- | --- | --- | --- |
|  | *Exp1* | *Exp2* | *Exp3* | *Exp1* | *Exp2* | *Exp3* |
| G3 Hemocytes | 1.00 | 1.00 | 1.00 | 1.00 | 1.00 | 1.00 |
| L3-5 Hemocytes | 2.16 | 3.64 | 2.93 | 7.36 | 4.59 | 6.97 |

Exp, experiment
