# Supplementary material for: The JNK Pathway Is a Key Mediator of Anopheles gambiae Antiplasmodial Immunity
Source: PLoS Pathog. 2013 Sep 5;9(9):e1003622. doi: 10.1371/journal.ppat.1003622 (PMC3764222; doi:10.1371/journal.ppat.1003622)
Supplement: Table S10 — Summary of oocyst data for all L3–5 infections. (DOCX) [file ppat.1003622.s016.docx]

**Table S10: Summary of oocyst data for all L3-5 infections**

| RNAi | *Exp1* | | | *Exp2* | | | *Exp3* | | | Combined | | | | | |
| --- | --- | --- | --- | --- | --- | --- | --- | --- | --- | --- | --- | --- | --- | --- | --- |
|  | N | Med  Live | Med  Mel | N | Med  Live | Med  Mel | N | Med  Live | Med  Mel | N | Med  Live | Med  Mel | M-W,  Live to Mel | Oocysts  mel (%) | Prev  (%) |
| LacZ | 18 | 0 | 33.5 | 28 | 0 | 42 | 21 | 0 | 19 | 67 | 0 | 32 | <0.0001 | 99.6 | 0 |
| JNK | 15 | 11 | 12 | 21 | 11 | 7 | 14 | 1 | 12 | 50 | 6 | 10.5 | 0.7192 | 32 | 70 |

Exp, experiment; Mel, melanized; Med, median; Prev, prevalence; M-W, Mann-Whitney
