# Supplementary material for: The JNK Pathway Is a Key Mediator of Anopheles gambiae Antiplasmodial Immunity
Source: PLoS Pathog. 2013 Sep 5;9(9):e1003622. doi: 10.1371/journal.ppat.1003622 (PMC3764222; doi:10.1371/journal.ppat.1003622)
Supplement: Table S11 — Primers used for dsRNA templates and silencing validation/real-time PCR. (DOCX) [file ppat.1003622.s017.docx]

**Table S11. Primers Used for dsRNA Templates and Silencing Validation/Real-Time PCR**

| Gene | AGAP ID | Primer Name | Primer Use | Primer Sequence |
| --- | --- | --- | --- | --- |
| JNK | AGAP009461 | JNKRNAiF | dsRNA from cDNA | TAATACGACTCACTATAGGGTGTTCCCCGGTACAGATCAT |
|  |  | JNKRNAiR | dsRNA from cDNA | TAATACGACTCACTATAGGGGTCGAACCACACGTTGATGT |
|  |  | JNKVerF | qPCR | TGCCAGGTCATACAGATGGA |
|  |  | JNKVerR | qPCR | CCCAAAGTCGAGGATTTTCA |
| Hep | AGAP001867 | HepRNAiF | dsRNA from cDNA | TAATACGACTCACTATAGGGAGCAGCAGCATTATGTGTCG |
|  |  | HepRNAiR | dsRNA from cDNA | TAATACGACTCACTATAGGGAGGTGACGGGGAGGTAGAGT |
|  |  | HepVerF | qPCR | ACTCTACCTCCCCGTCACCT |
|  |  | HepVerR | qPCR | GGATTTGCAGTCCCACTCAT |
| Jun | AGAP006386 | JunRNAiF | dsRNA from cDNA | TAATACGACTCACTATAGGGCGGTACAACACGTCCATCAC |
|  |  | JunRNAiR | dsRNA from cDNA | TAATACGACTCACTATAGGGAGGGTGACACTGTTGGCACT |
|  |  | JunVerF | qPCR | AGGGCAAGTTTTGAATGCAC |
|  |  | JunVerR | qPCR | CACGCACTTTCTCCCTTTGT |
| Fos | AGAP001093 | FosRNAiF | dsRNA from cDNA | TAATACGACTCACTATAGGGGTCCACACAAGCCCTCAAAT |
|  |  | FosRNAiR | dsRNA from cDNA | TAATACGACTCACTATAGGGTCTGCGTTGCACGTAGATTC |
|  |  | FosVerF | qPCR | CCGTTCAACCGTTTTCAAGT |
|  |  | FosVerR | qPCR | ACGAATCCTGCTTGGTATGG |
| Puc | AGAP004353 | PucRNAiF | dsRNA from cDNA | TAATACGACTCACTATAGGGACTGTCAGGCTGGCATTAGC |
|  |  | PucRNAiR | dsRNA from cDNA | TAATACGACTCACTATAGGGGTTGAGCGGGTTGAACTTG |
|  |  | PucVerF | qPCR | CGCCCTGCCTGATGCTACGG |
|  |  | PucVerR | qPCR | ACGTGTGATGCTGGGTGCGA |
| Nox5 | AGAP008072 | Nox5RNAiF | dsRNA from plasmid | TCGTACGAATGGTCGAGTGA |
|  |  | Nox5RNAiR | dsRNA from plasmid | CCAAACTGGTCGCACTTGTA |
|  |  | Nox5VerF | qPCR | TCATGCATCGCTACTGGAAG |
|  |  | Nox5VerR | qPCR | CCAGAAAAGTCCACCTTGG |
| Hpx2 | AGAP009033 | Hpx2RNAiF | dsRNA from cDNA | TAATACGACTCACTATAGGGACGACGACGGTGTGTACAAG |
|  |  | Hpx2RNAiR | dsRNA from cDNA | TAATACGACTCACTATAGGGATACTCGGCCGAATCGAAC |
|  |  | Hpx2VerF | qPCR | CCGCTTCTACAACACGATGA |
|  |  | Hpx2VerR | qPCR | CGACCAGATGGGCAAGTAT |
| Tep1 | AGAP010815 | Tep1RNAiF | dsRNA from plasmid | TTTGTGGGCCTTAAAGCGCTG |
|  |  | Tep1RNAiR | dsRNA from plasmid | ACCACGTAACCGCTCGGTAAG |
|  |  | Tep1VerF | qPCR | ATACGGATCTCAGCTATACCAAATCG |
|  |  | Tep1VerR | qPCR | TGCGGGCCTTTATGAGAAAA |
|  |  | Tep1G3/L35F* | qPCR | Cgatgatgttgaacggacac |
|  |  | Tep1G3/L35R* | qPCR | Cacaaacgacagcagagcat |
| Fbn9 | AGAP011197 | Fbn9RNAiF | dsRNA from cDNA | TAATACGACTCACTATAGGGCCAAGATGTCGGGCAAGTAT |
|  |  | Fbn9RNAiR | dsRNA from cDNA | TAATACGACTCACTATAGGGTTGTGGTACGTCAGCGAGTC |
|  |  | Fbn9VerF | qPCR | CCAAGATGTCGGGCAAGTAT |
|  |  | Fbn9VerR | qPCR | CACCCTTAAACCGATGCTGT |
| APL1A | AGAP007036 | APL1AVerF  APL1AVerR | qPCR  qPCR | GTAAACGCGCTGAACTGCGGTGCAGC  TCTGGTCTTGTATGTACAATGGAACC |
| APL1C | AGAP007033 | APL1CVerF  APL1CVerR | qPCR  qPCR | CTGCTGCAGGGGCTACACGCC  GGCCCAAGTAACATCATACAC |
| LRIM1 | AGAP006348 | LRIM1VerF  LRIM1 VerR | qPCR  qPCR | CATCCGCGATTGGGATATGT  CTTCTTGAGCCGTGCATTTTC |
| AgS7 | AGAP010592 | AgS7F | qPCR | AGAACCAGCAGACCACCATC |
|  |  | AgS7R | qPCR | GCTGCAAACTTCGGCTATTC |
| LacZ | n/a | LacZRNAiF | dsRNA from plasmid | GAGTCAGTGAGCGAGGAAGC |
|  |  | LacZRNAiR | dsRNA from plasmid | TATCCGCTCACAATTCCACA |

*Tep1 primers used for Fig 3 data contain mismatches in L3-5 sequence. New primers were designed in conserved regions for data comparing expression in S vs. R. All other primers used for S and R comparisons share identical sequence between strains.
